# Cell-free synthesis of the hirudin variant 1 of the blood-sucking leech *Hirudo medicinalis*

**DOI:** 10.1038/s41598-020-76715-w

**Published:** 2020-11-13

**Authors:** Doreen A. Wüstenhagen, Phil Lukas, Christian Müller, Simone A. Aubele, Jan-Peter Hildebrandt, Stefan Kubick

**Affiliations:** 1 Fraunhofer Institute for Cell Therapy and Immunology (IZI), Branch Bioanalytics and Bioprocesses Potsdam-Golm (IZI-BB), 14476 Potsdam, Germany; 2 Animal Physiology and Biochemistry, Zoological Institute and Museum, University of Greifswald, 17489 Greifswald, Germany; 3 Faculty of Health Sciences, Joint Faculty of the Brandenburg University of Technology Cottbus – Senftenberg, The Brandenburg Medical School Theodor Fontane and the University of Potsdam, 16816 Neuruppin, Germany

**Keywords:** Expression systems, Cardiovascular biology, Proteases

## Abstract

Synthesis and purification of peptide drugs for medical applications is a challenging task. The leech-derived factor hirudin is in clinical use as an alternative to heparin in anticoagulatory therapies. So far, recombinant hirudin is mainly produced in bacterial or yeast expression systems. We describe the successful development and application of an alternative protocol for the synthesis of active hirudin based on a cell-free protein synthesis approach. Three different cell lysates were compared, and the effects of two different signal peptide sequences on the synthesis of mature hirudin were determined. The combination of K562 cell lysates and the endogenous wild-type signal peptide sequence was most effective. Cell-free synthesized hirudin showed a considerably higher anti-thrombin activity compared to recombinant hirudin produced in bacterial cells.

## Introduction

Hirudin is a secretory protein produced in the salivary gland cells of sanguivorous leeches of the genus *Hirudo*^1,2^ and one of the most effective natural thrombin inhibitors. First described by Haycraft in 1884, it took almost 80 years until Markwardt et al. were able to successfully isolate and purify hirudin from crude extracts of *Hirudo medicinalis*^1–4^. However, extraction of native hirudin was very time consuming, costly and inefficient and required sacrificing a large number of leeches^2,5,6^. The availability of mRNA/cDNA sequence data of hirudin completely altered the situation and enabled the production of large amounts of recombinant hirudin in prokaryotic and eukaryotic expression systems. Bacterial expression systems include *Escherichia coli* (*E. coli*)^7–10^, *Bacillus subtilis*, *Lactococcus lactis* and *Streptomyces lividans*^11,12^, whereas in fungi hirudin could be successfully synthesized in *Acremonium chrysogenum*, *Ogataea angusta*, *Pichia pastoris* and *Saccharomyces cerevisiae*^13–16^, respectively. Eukaryotic expression systems, beside fungi, include insect cell lines, Chinese hamster ovary (CHO) cells, mouse fibroblast cells, porcine endothelial cells and pituitary secretory cell lines^17–19^. In addition, a cell-free synthesis approach with rabbit reticulocyte lysate was described as well^20^. Commercially available hirudin variants like Desirudin and the discontinued Lepirudin are nowadays expressed in *Saccharomyces cerevisiae*^21–23^, whereas for research applications usually protocols based on *E. coli* or *Pichia pastoris* are applied^7,23–25^.

The expression of recombinant hirudin in bacteria or yeast usually results in products with a lower activity compared to leech-derived hirudin. One explanation for this phenomenon is the absence of sulfatation at the amino acid residue Tyr-63 (Tys-63) or other post-translational modifications like glycosylations^26–29^. In only a few reports the successful synthesis of sulfo-hirudin using chemical synthesis, expression in baby hamster kidney (BHK) cells or even in *E. coli* cells was described^28,30,31^.

Misfolding of recombinant proteins in general and hirudin in particular is a major concern as well. In addition to the lower activity, misfolded proteins applied in patients may cause or amplify unwanted immunogenic reactions or other side effects^32^. Misfolding mostly happens during inclusion body formation, which is typically a consequence of high yield expression in bacterial systems^33–36^. Another important aspect of recombinant protein expression especially for medical applications is the presence of endotoxins or other byproducts in the final extracts. Such contaminations have to be detected and carefully removed prior to application^37^. Taken together, the synthesis of sufficient amounts of native (sulfo) hirudin for research or clinical applications is still a challenging task.

Cell-free protein synthesis approaches might be a promising alternative to the conventional methods described above. In cell-free systems, protein synthesis is based on the presence of the translational apparatus of the cells only, while other cell components like the nuclei, mitochondria or the outer membrane are removed^38^. By choosing specific lysates, unwanted byproducts like endotoxins can be easily avoided. In eukaryotic cell lysates, the complex translational characteristics remain intact and thus the chance of correct protein folding and posttranslational modifications like sulfatation and glycosylation is significantly enhanced^39^. During the lysate production process, endogenous microsomal vesicles based on the endoplasmic reticulum (ER) are obtained. The native translocon remains in an active state and proteins with signal sequences can be translocated into the lumen of the microsomes. Furthermore, endogenous disulfide isomerases are located in the lumen of the microsomes and N-glycosylation (core) also takes place here^40,41^. These are important prerequisites for correctly folded and active proteins.

In the present study we describe a new experimental approach to the cell-free synthesis of hirudin variant 1 (HV1 or hirudin-VV) of *Hirudo medicinalis*. The efficiencies of three different eukaryotic cell-free systems based on *Spodoptera frugiperda* (*Sf*21), Chinese hamster ovary (CHO) and human K562 cell lines and the effects of two different cleavable signal peptide sequences on the synthesis and correct processing of mature hirudin were examined. In vitro assays were performed for the functional characterization of cell-free synthesized hirudin in comparison to hirudin that has been conventionally synthesized in bacterial expression systems. Our data indicate a considerably higher anti-thrombin activity of the cell-free produced hirudin. Given the scalability of cell-free protein synthesis, which has already been demonstrated in *E. coli* cell-free systems^42,43^, this approach could be a promising alternative for the production of highly active hirudin (and other protein drugs with complex molecular structures).

## Results

### Cell-free synthesis of hirudin in three different eukaryotic cell lysates

We have previously demonstrated the performance of cell-free protein synthesis systems based on translationally active *Sf*21, CHO and K562 cell lysates^41,44–47^. All three cell-free lysate systems contain endogenous microsomes derived from the ER. An advantage of these microsomes is the co-translational translocation of proteins harbouring a signal peptide into the lumen of the microsomes. Thereby, subsequent post-translational modifications such as cleavage of signal peptides, formation of disulfide bridges, *N*-glycosylation etc. takes place as in living cells^39^. Chaperones, which play an important role in protein folding are also present in the microsomes. In order to obtain correctly folded and thus active hirudin, we have decided to use the potential of the microsomes, since hirudin is very sensitive to any modifications at the N-terminus and also at the C-terminus, such as the addition of a His-tag for protein purification^48^.

**Figure 1:**
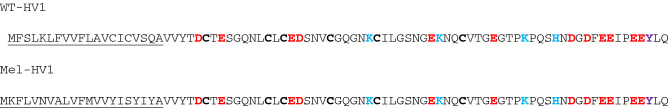
Sequence comparison of hirudin with its endogenous signal peptide (WT-HV1) or with the melittin signal (Mel-HV1). The signal sequence is underlined, while the conserved six cysteines are bolded, basic amino acids are indicated as blue, acid amino acids as red and tyrosine at position 63 as purple. The sequence is given in one-letter code according to IUPAC.

We have constructed two different vectors to express hirudin HV1. The first construct contained the endogenous wild-type signal peptide sequence of hirudin (wild-type hirudin, WT-HV1) and the second contained hirudin fused to the heterologous signal peptide sequence of melittin (Mel-HV1) instead of the endogenous sequence (see Fig. 1).

In a first evaluation (“proof of concept”) only WT-HV1 was tested for expression and processing in three different cell lysate systems, the insect cell line *Sf*21, the human cell line K562 and the hamster cell line CHO-K1. Total protein yields and the secretion efficiencies were determined and quantified by scintillation and autoradiography. The term secretion efficiency refers to the processed protein that was translocated into the lumen of the ER-based microsomes and released from the microsomes using a mild detergent after completion of protein synthesis (see preparation of SN2 in methods section). For better comparability all calculations were normalized to the molecular mass of mature hirudin without signal peptide (7 kDa).

**Figure 2:**
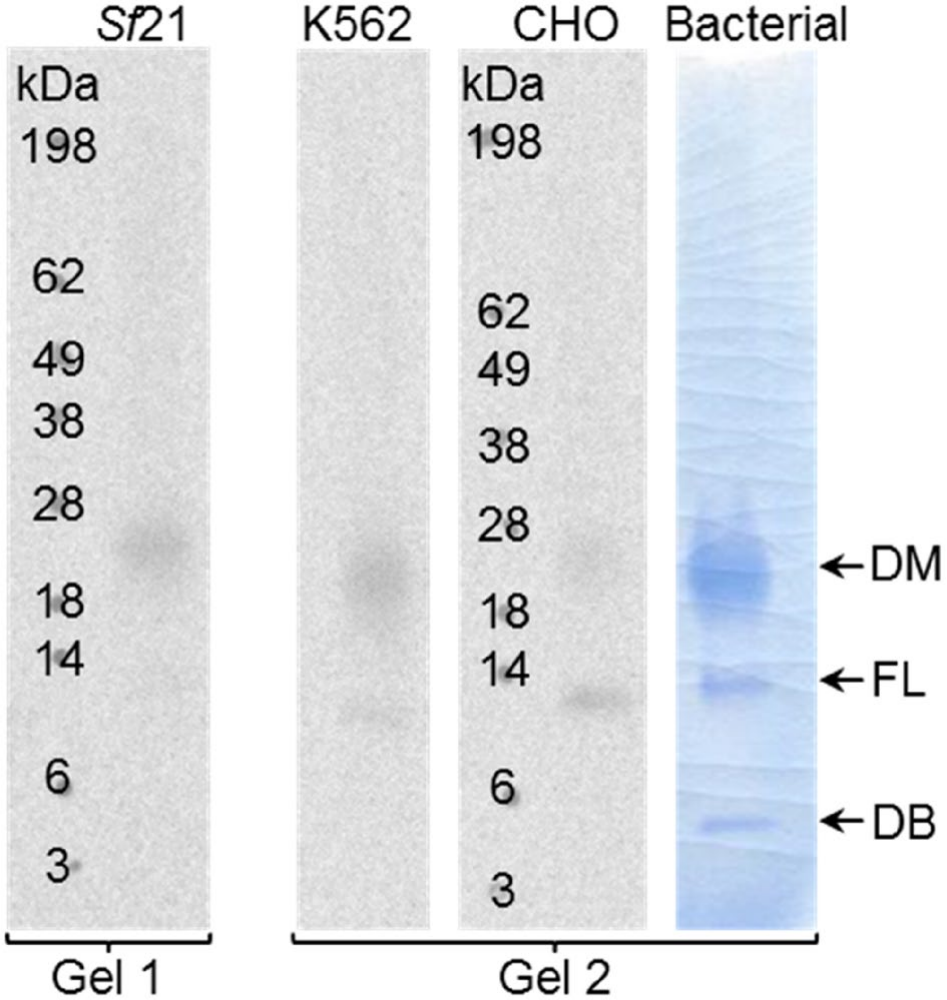
Autoradiography of translocated and released WT-HV1 (SN2 fraction) in three different eukaryotic cell-free systems derived from *Sf*21, K562 and CHO cells, respectively. For comparison, the Coomassie staining of bacterial hirudin is shown, which was analyzed on the same SDS gel. All three cell-free systems displayed two bands indicating full length hirudin (FL) at approx. 13 kDa and a putative dimer of hirudin (DM) at approx. 26 kDa. The same protein bands can be detected in the bacterial hirudin. Additional debris (DB) could be observed between 3 and 6 kDa. The shown image consists of two different gels, which contained the used protein ladder and HV1 from *Sf*21-lysate (Gel1) and the protein ladder together with HV1 from K562-lysate, from CHO-lysate and the bacterial hirudin (Gel2). The autoradiographs are based on the SDS gels.

As shown in Fig. 2, WT-HV1 was successfully expressed in all three cell free expression systems and mainly appeared as a dimer (26 kDa) or monomers (13 kDa) on autoradiographs of protein blots. The relative abundances of monomers and dimers were slightly different with monomers being the major forms in cell free synthesis reactions using CHO extracts. Dimers appeared to be more abundant than monomers in reactions using *Sf*21 or K562 extracts. Both, the dimer and the monomer were also detectable using Coomassie staining of bacterial expressed hirudin (see Fig. 2).

However, both the total protein yield, the amount of secreted hirudin (final processed protein without the signal peptide; SN2 fraction) and the secretion efficiency (ratio between total protein yield and released protein) markedly differed (Table 1). The highest total protein yield with 1065 nmol/l (7.5 µg/ml) was obtained in the K562 cell-free system with a secretion amount of translocated and released hirudin of about 50 nmol/l (0.4 µg/ml). The CHO cell-free system was less productive in terms of total yield (211 nmol/l; 1.5 µg/ml), but it resulted in an equal amount of secreted hirudin (about 51 nmol/l or 0.4 µg/ml, final concentration) in comparison to the K562 system. Using the *Sf*21 cell-free system a total protein yield of 838 nmol/l (5.9 µg/ml) and a secreted amount of hirudin of about 70 nmol/l (0.5 µg/ml) was obtained.

**Table 1:** Synthesis of wild-type hirudin (WT-HV1) in three different eukaryotic cell-free systems: total protein yield and secreted/released amount of hirudin (SN2 fraction) in nmol/l and µg/ml; ratio between total protein yield and secreted/released amount of hirudin represents the secretion efficiency.

	K562	*Sf*21	CHO
Total protein yield	1065 nmol/l	838 nmol/l	211 nmol/l
	7.5 µg/ml	5.9 µg/ml	1.5 µg/ml
Secreted amount	50 nmol/l	70 nmol/l	51 nmol/l
	0.4 µg/ml	0.5 µg/ml	0.4 µg/ml
Ratio	4.7%	8.4%	24.1%

Determination was performed by double determination (n = 2).

### Anti-coagulatory activity of cell-free synthesized hirudin

**Figure 3:**
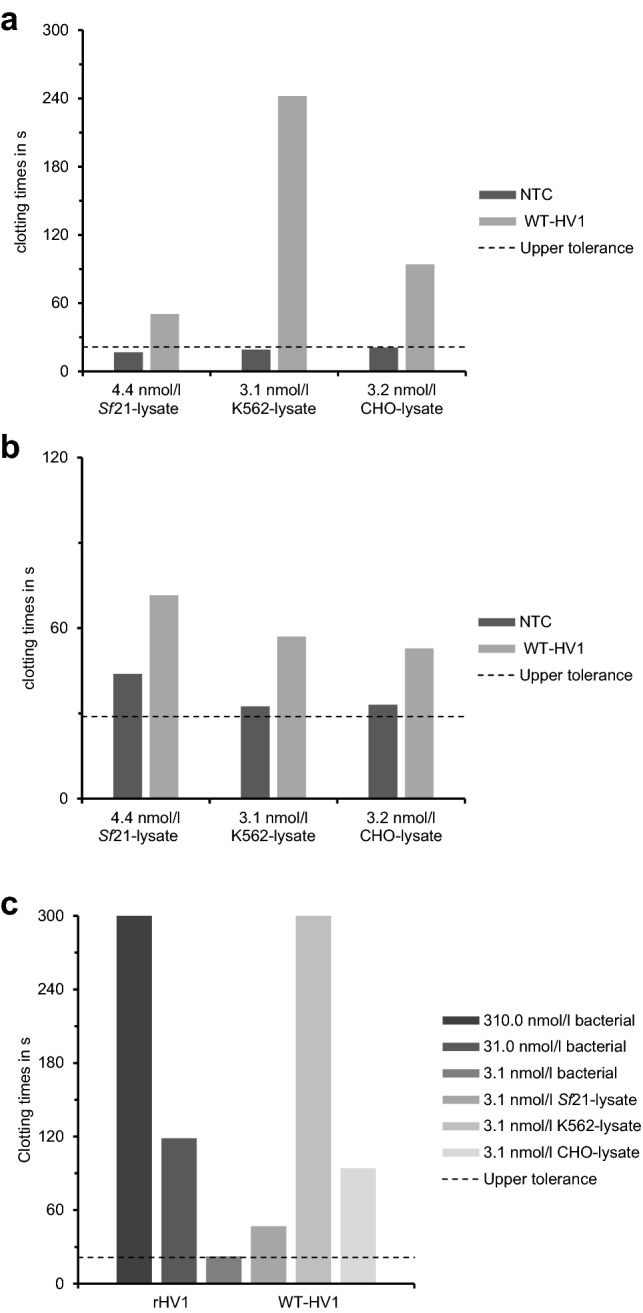
Anti-coagulatory potencies of translocated and released WT-HV1 samples (SN2) synthesized in *Sf*21, K562 and CHO cell-free systems tested in the thrombin time assay (**a**), activated partial thromboplastin time assay (**b**) and anti-coagulatory potencies of equimolar amounts of HV1 synthesized by bacterial expression (rHV1) and synthesized in *Sf*21, K562 and CHO cell-free systems (WT-HV1, SN2) determined by the thrombin time test assay (**c**). Final concentrations are given in nmol/l. For the equimolar measurement samples were tested at a final concentration of 3.1 nmol/l. rHV1 was additionally tested at a concentration of 31.0 nmol/l. Data for the activity of rHV1 at a concentration of 310 nmol/l were taken from Müller et al.^71^. For determination of clotting times double determination was used as “proof of principle” (n = 2).

The processed and released hirudin preparations (SN2 fractions) obtained from all three cell-free systems were independently tested for their anti-coagulatory activities in two different coagulation assays (“proof of principle”): the activated partial thromboplastin time (aPTT) test as well as the thrombin time (TT) test. Whereas the aPTT test is used to determine the functionality of the intrinsic and common pathways of the coagulation cascade, the TT is applied to specifically test for activity of thrombin. 10 µl of each SN2 fraction were added to the respective coagulation assays and the time until onset of coagulation was measured. SN2 fractions of the respective no-template control reactions (NTC) served as negative controls. In all cases, SN2 fractions containing hirudin clearly prolonged the coagulation time in the TT test, whereas the NTC did not negatively affect the coagulation times (Fig. 3a). The anti-coagulatory effect caused by hirudin was most pronounced for hirudin synthesized in the human K562 cell-free lysate system (clotting time about 242 s), whereas the expression of hirudin in the CHO (94 s) and *Sf*21 cell-free lysate system (50 s) were less effective. The results for the aPTT assays were similar, apart from the fact that all hirudin-containing SN2 fractions were evenly increasing aPTT while the NTC fractions prolonged the coagulation times just marginally above the upper tolerance limit at 28.9 s (Fig. 3b).

### Comparison of cell-free vs. bacterially synthesized hirudin samples

So far, variants of both hirudins and hirudin-like factors were produced and purified solely from *E. coli*-based expression systems in our lab. To directly compare the anti-coagulatory activities of both bacterially (rHV1) and cell-free synthesized HV1 samples, we performed thrombin time tests with equimolar concentrations (3.1 nmol/l) of each sample. In previous investigations, rHV1 was routinely tested in concentrations of 3100 and 310 nmol/l, respectively. Thus we decided to test also rHV1 at concentrations of 31 nmol/l and 3.1 nmol/l to cover the wider range of four orders of magnitude. Compared with rHV1 expressed in bacteria the WT-HV1 expressed with extracts of *Sf*21 lysate was at least twice as effective in elongation of TT (Fig. 3c). WT-HV1 expressed using extracts of CHO-lysate was even more efficient and elongated TT approximately ninefold more efficiently compared with protein expressed in bacteria. The most potent WT-HV1 was that expressed using extracts of K562 cells. This variant was approximately 100 times more effective as an anti-coagulant than rHV1 expressed in bacteria (Fig. 3c).

### Comparison of cell-free synthesis of WT-HV1 and Mel-HV1

We also synthesized and functionally tested Mel-HV1. The signal peptide sequence of melittin is commonly used for labelling, subsequent processing and highly efficient secretion of proteins in heterologous expression systems^49^. For cell-free protein synthesis, especially for efficient translocation of proteins into the lumen of microsomes, the use of the melittin signal peptide sequence has already been described and is firmly established in our lab^38–40,50^. Based on the results of the evaluation of the synthesis of WT-HV1 in the three eukaryotic cell-free lysate systems, only the human K562 system was used for this study. The resulting total protein yields and secreted/released amounts of WT-HV1 and Mel-HV1 are listed in Table 2. The total yield of WT-HV1 (1245 nmol/l or 8.8 µg/ml) was approximately the same compared to the first synthesis reaction, but the secreted/released amount of hirudin (158 nmol/l or 1.1 µg/ml) and hence the secretion efficiency (12.7%) was nearly three-times higher. In contrast, both the total protein yield of Mel-HV1 (6150 nmol/l or 43.2 µg/ml) and the secreted/released amount of hirudin (510 nmol/l or 3.6 µg/ml) were considerably higher compared to WT-HV1, whereas the secretion efficiency (8.3%) was slightly lower.

**Table 2:** Cell-free synthesis of WT-HV1 and Mel-HV1 in K562 cell lysate system: total protein yield and secreted/released amount of hirudin (SN2 fraction) in nmol/l and µg/ml; ratio between total protein yield and secreted/released amount of hirudin represents the secretion efficiency.

	Total yield	Secreted amount	Ratio (%)
WT-HV1	1245 nmol/l	158 nmol/l	12.7
	8.8 µg/ml	1.1 µg/ml	
Mel-HV1	6150 nmol/l	509 nmol/l	8.3
	43.2 µg/ml	3.6 µg/ml	

Determination was performed by double determination (n = 2).

### Processing of hirudin samples containing either the endogenous wild-type or the heterologous melittin signal peptide

**Figure 4:**
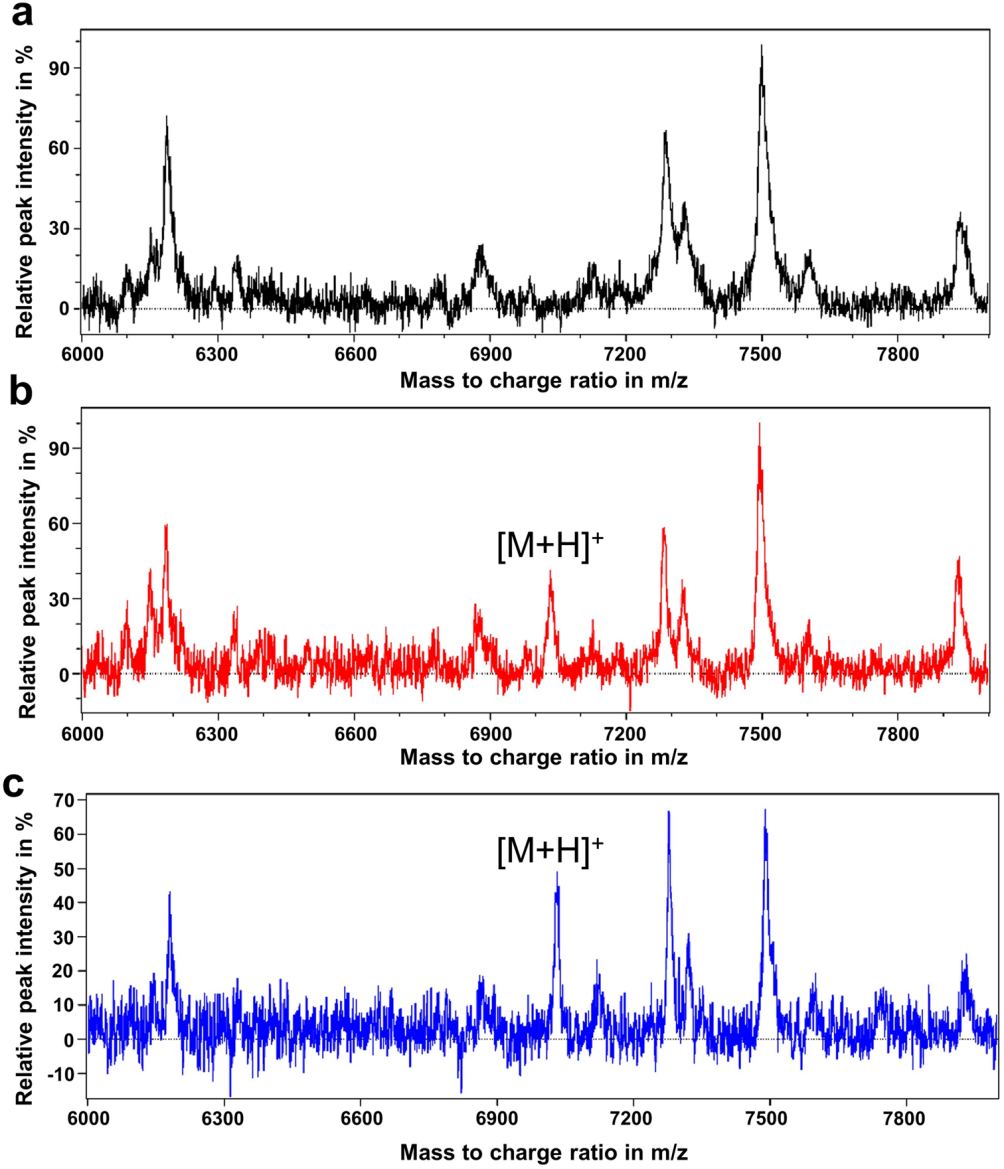
Mass spectrometric analyses of SN2 fractions derived from K562 cell-free system expressing either no hirudin (NTC as negative control) (**a**), hirudin HV1 containing the endogenous wild-type (**b**) or the heterologous melittin signal peptide (**c**). Each sample was analysed by three independent measurements. The predicted AVG of HV1 is 7026.6 Da. The additional peaks indicated by arrows represent masses of 7025.6 ± 6.0 Da (**b**) or 7027.5 ± 5.0 Da (**c**), respectively.

As hirudins still containing their signal peptides are not able to function as anti-coagulants^17,51^, the high anti-coagulatory and anti-thrombin potencies of WT-HV1 variants generated by cell-free protein synthesis indicates that the endogenous wild-type signal peptide had been removed by the signal peptidase present in all three cell lysates. To verify the correct processing of both the endogenous wild-type and the heterologous melittin signal peptides, we analysed the SN2 fractions of WT-HV1, Mel-HV1 and NTC, based on the cell-free synthesis in the K562 cell lysate system, by MALDI-TOF. The spectra of both WT-HV1 and Mel-HV1 SN2 fractions revealed the presence of additional peaks at a molecular mass of about 7026 Da compared to the spectrum of NTC SN2 fraction (Fig. 4). The predicted molecular mass of HV1 is 7026.6 Da, which is almost identical to the observed values (arrows). These data strongly support the assumption that not only WT-HV1, but also Mel-HV1 is correctly processed during cell-free synthesis in K562 cell lysates.

### Anti-thrombin activities of WT-HV1 and Mel-HV1 synthesized in the human K562 cell-free system

**Figure 5:**
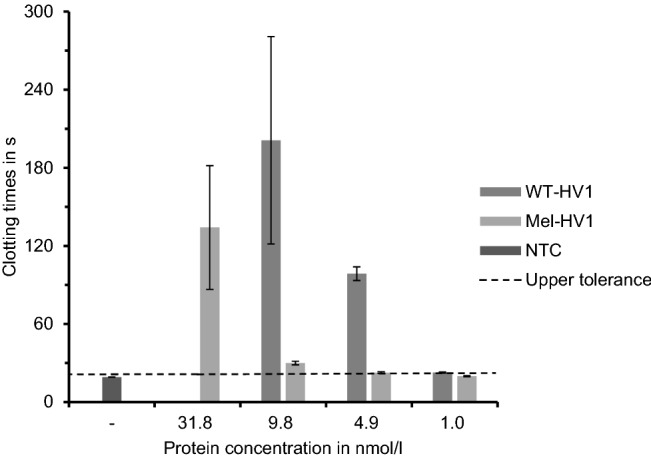
Anti-coagulatory activities of SN2 fractions containing either WT-HV1 or Mel-HV1 synthesized in the K562 cell-free system as determined by the thrombin time test assay. A cell-free reaction without synthesized hirudin was used as a negative control (NTC). Final concentrations of test proteins are given in nmol/l (n = 3).

To test the anti-coagulatory potency of WT-HV1 or Mel-HV1 we performed comparative thrombin time test assays with concentrations of 9.8 nmol/l (highest achievable concentration of WT-HV1), 4.9 nmol/l and 1 nmol/l of each of the inhibitors, respectively. For Mel-HV1, we additionally tested the highest achievable concentration of 31.8 nmol/l. The results are summarized in Fig. 5 and clearly show that the anti-coagulatory potency of WT-HV1 was much more pronounced (at least by a factor of 10) compared with that of Mel-HV1. Additionally, at the highest concentration of 31.8 nmol/l, Mel-HV1’s anti-thrombin activity reached just the same level as the bacterially expressed rHV1 at nearly the same concentration (see Fig. 3c). A further comparison between the SN1-fraction of the HV1 showed lower influence on clotting times, while concentration of the SN1-fraction was at least twice as high as the SN2-fraction (see Supplementary Files).

### Posttranslational modifications of hirudin synthesized in the human K562 cell-free lysate system

**Figure 6:**
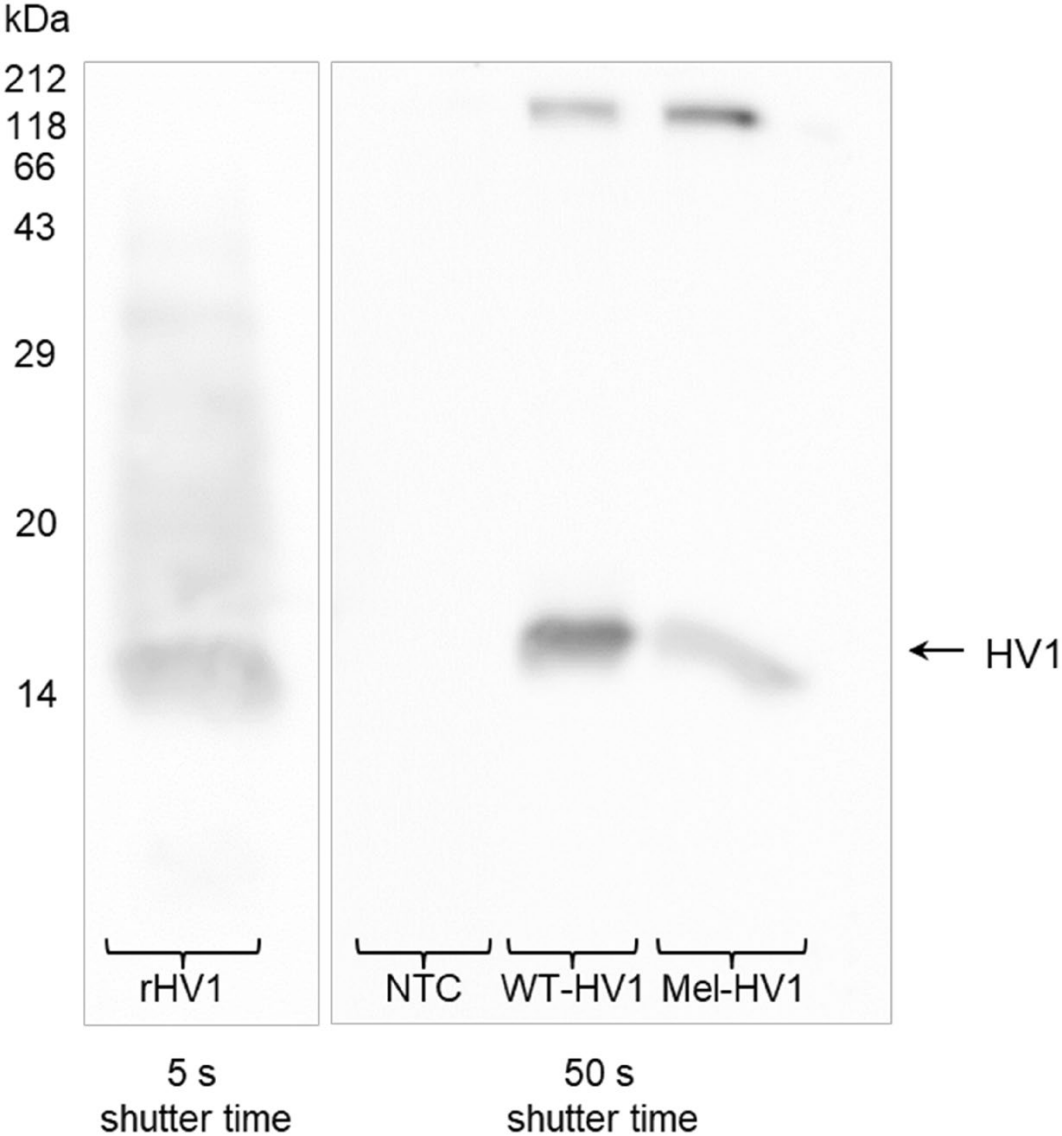
Western-blot analysis of different hirudin preparations. rHV1: bacterially expressed hirudin; WT-HV1 and Mel-HV1: cell-free synthesized in K562 lysate; NTC: no template control (cell-free reaction without synthesized hirudin). The SN2-fractions of the microsomes were used for all cell-free produced protein samples. The image of the Western Blot was cropped from the same Western Blot exposed by two different shutter times (5 s and 50 s), to avoid overexposure as well as faded bands. The corresponding blot areas were delineated by boxes. The images of all exposure times are summarized in the Supplementary Material.

The hirudin variants of the European medicinal leeches contain a tyrosine residue at position 63 (Tyr-63 in HV1 and HV2) or 64 (Tyr-64 in HV3) at the C-termini (see above). The respective residues are sulfated, but only in hirudins that are purified directly from salivary glands of leeches. Except from only very few counterexamples^30,31^, neither hirudins expressed in eukaryotic nor in bacterial systems contain this modification. The sulfatation of Tyr-63/Tyr-64 enhances the negative charge of the C-terminal tail of hirudin and stabilizes its interaction with thrombin, thereby promoting the inhibitory activity^52^. To evaluate the putative post-translational modification of cell-free synthesized hirudins we performed Western blot analysis using antibodies that were raised against either hirudin (whole protein antibody) or sulfo-tyrosine. Bacterially expressed rHV1 served as a control for whole protein detection, NTC SN2 fraction as the negative control. The anti-hirudin antibody detected its target in SN2 fractions of WT-HV1 and Mel-HV1, but not in NTC (Fig. 6). However, no evidence could be found for sulfatation of Tyr-63/64 in any of the fractions (data not shown).

## Discussion

The present study describes the successful synthesis of hirudin variant HV1 in three different eukaryotic cell-free systems. The impacts of either the endogenous wild-type or the heterologous melittin signal peptides on synthesis, processing and secretion of hirudin was evaluated. Finally, the anti-coagulatory activity of different cell-free synthesized hirudin samples was determined.

The large-scale synthesis and purification of hirudin for scientific, commercial and medical purposes remains a challenging task^16,23^. Preparations of hirudin from whole leeches may lead to ethical concerns (death of innumerable animals) and/or limitations due to the collapse of natural populations^2,53^. The biotechnological production of hirudin in bacteria, yeast and animal cells is hence an attractive alternative^16^. However, there are major drawbacks and limitations in heterologous expression, too^54^. Putative contaminations with bacterial endotoxins in animal cells may serve as examples^37^. Cell-free synthesis of proteins in general and hirudin in particular has the potential to avoid most of the problems mentioned above. The efficiency of cell-free protein synthesis largely depends on the appropriate expression system with the matching vector construct and the background of the corresponding subcellular machinery as the main components^44,55^. We have examined lysates of three different eukaryotic cell lines, namely human K562, insect *Sf*21 and CHO-K1, for the expression of hirudin variant 1 (HV1). In all three eukaryotic cell-free systems, hirudin was successfully synthesized and secreted. However, there were obvious differences between the individual cell lysates. Lysate derived from the human cell line K562 was best suited in terms of total yield and amount of secreted protein, whereas the CHO lysate displayed the highest secretion efficiency. One might expect that the lysate derived from the cell line *Sf*21 would have performed best due to the closer phylogenetic relationship of *Insecta* and *Annelida*. However, this was the case only for the secreted/released amount of hirudin, but neither for the total yield nor the ratio between total yield and secreted/released protein (see Table 1).

The signal peptide sequence of the honey bee toxin melittin is often used for the efficient secretion of proteins in heterologous cell-based and cell-free expression systems^49,56,57^. We have compared the synthesis and secretion of hirudin containing either its endogenous wild-type signal peptide (WT-HV1) or the heterologous melittin signal peptide (Mel-HV1) in K562 cell lysates. Both the total protein yield and the amount of secreted hirudin were considerably higher for Mel-HV1. The secretion efficiency, however, was slightly lower. These observations are in good agreement with previous reports^38,56^. The total protein yield of synthesized hirudin in the translation reaction was about 6150 nmol/l (43.2 µg/ml) for Mel-HV1 and 1245 nmol/l (8.8 µg/ml) for WT-HV1. Both quantities are in the typical range of cell-free synthesized luciferase^38,50^ and membrane proteins^58^. Taken together, we successfully conducted cell-free synthesis of hirudin both with its endogenous wild-type signal peptide (WT-HV1) as well as with the heterologous melittin signal peptide (Mel-HV1).

The anti-coagulatory and anti-thrombin potencies of hirudin can be determined and quantified by several coagulation assays, among them the aPTT and the TT tests^59^. Since we did not purify hirudin from the cell lysates, we performed all assays with the respective SN2 fractions that contained proteins that were translocated into the lumen of the ER-based microsomes during the cell free synthesis reaction and subsequently released by treatment of the microsomes with the mild detergent DDM. In all assays, the hirudin-containing SN2 fractions considerably prolonged the coagulation times in comparison to the respective NTC samples (Fig. 3). Hence, all WT-HV1 SN2 fractions contained biologically active hirudin. However, there were differences between the lysates. The K562 cell lysate displayed by far the strongest impact on the coagulation time in the TT test, despite the lowest concentration of secreted hirudin (3.1 nmol/l). In contrast, the *Sf*21 SN2 fraction contained the highest concentration of secreted hirudin (4.4 nmol/l), but displayed only a comparably low anti-coagulatory potency (see Fig. 3a,b). Differences in the yield of active protein between lysates of the three cell lines have already been described for the firefly luciferase^44^. In addition to that clotting times of the bacterial expressed hirudin variant rHV1 were in good accordance with respective values of the commercially available lepirudin as described in previous studies^48,60,61^. Based on this data the anti-thrombin activity of hirudin in all three cell lysates was additionally compared to the activity of the bacterially expressed rHV1. Anyway, in respect to the concentration of the SN2 fractions a concentration range of 3100 nmol/l to 3.1 nmol/l was tested for rHV1, which included 100–1000-fold amounts of rHV1 which were usually tested. As can be seen in Fig. 3c, all cell-free synthesized hirudins seem to display higher anti-thrombin potencies compared to rHV1 expressed in bacteria. Again, hirudin synthesized in the K562 cell lysate had the strongest impact.

The differences of anti-coagulatory potencies among the cell-free synthesized hirudin samples and, even more important, in comparison to rHV1 expressed in bacteria might be explained by differences in folding of the mature protein. Activity of hirudin critically depends on the correct formation of three disulphide bonds within the central globular domain^15,29^. It seems very likely that the cellular machinery of eukaryotic cells performs better in expression and handling of heterologous proteins of eukaryotic origin in comparison to the bacterial cell machinery^54^. Moreover, protein misfolding and formation of inclusion bodies remain a serious problem and a technological challenge for the production of recombinant proteins in bacterial cells^33–35^. Cell-free protein synthesis could be a promising alternative harbouring the potential to overcome these problems. However, not all cell lysates are equally well suited for all target proteins^44,50^. Accordingly the selection of the appropriate cell-free system (e.g. K562 cell lysate in case of hirudin) and the optimization of the reaction conditions^45,50,62,63^ to the needs of the respective protein should be carried out in form of evaluation studies.

One parameter for optimization might be the choice of the appropriate signal peptide sequence. For secretory proteins, the signal peptide sequence of melittin (Mel-SP), a honey bee toxin, is quite regularly used and works well for a broad variety of proteins^49,56,58^. Interestingly, the Mel-SP did not improve our results for hirudin significantly. Mel-HV1 was synthesized in considerably larger amounts in the K562 cell-free system compared to WT-HV1 and the secretory amount was more than three times higher, too (see Table 2). The anti-thrombin potency of Mel-HV1, however, was lower compared to that of WT-HV1 (Fig. 5). At equimolar concentrations, WT-HV1 had a clearly detectable anti-coagulatory effect, whereas Mel-HV1 had almost none (at 4.9 nmol/l) or a very weak effect (at 9.8 nmol/l).

A possible explanation for the lower effectivity of Mel-HV1 compared to WT-HV1 as an anti-coagulant might be an incorrect processing of the Mel-SP. The activity of hirudin is negatively influenced by alterations of the N-terminal five amino acid residues^20,64^. However, the mass determinations of WT-HV1 and Mel-HV1 by MALDI-TOF analyses did not reveal any evidence for an incorrect processing of the signal peptides. Both spectra contained a clearly visible peak at a position that exactly corresponds to the expected molecular mass of hirudin. Alternatively, the lower potency of Mel-HV1 compared to that of WT-HV1 as an anti-coagulant may be explained by incorrect folding of the respective protein. Our data indicate that a heterologous signal peptide might somehow affect and even hamper the correct folding of a protein upon secretion. A similar observation on the influence of the endogenous signal peptide on the correct folding of a bacterial autotransporter has been made by other researchers^65^. The authors concluded that some types of signal peptides play major roles in preventing misfolding of mature proteins. Hence, possible negative effects of a heterologous signal peptide on the activity of the cell-free synthesized "protein of interest" should therefore be considered and, consequently, tested.

Hirudins of European medicinal leeches of the genus *Hirudo* are usually sulphated at tyrosine residues at positions 63 or 64, respectively. With only a very few exceptions^9,30,31^, hirudins of biotechnological origin do not contain the respective sulphates. In addition, hirudins of the Asian medicinal leech *Hirudinaria manillensis* are glycosylated as well^27,66^. Neither WT-HV1 nor Mel-HV1 displayed any signs of post-translational modifications like the addition of a sulphate groups or of carbohydrate residues (Figs. 4, 6).

## Conclusion

Hirudin is a drug of medical relevance in clinical use for decades^67,68^. So far, the biotechnological production of recombinant hirudin depends on either bacterial or yeast expression systems^16^. Both systems have major drawbacks in terms of putative contaminations and limitations in terms of yield of biologically active product^34,35,37^. In the present study, we investigated further promising ways to produce hirudin in its active form. The cell-free human K562 system in particular shows a high potential to produce active hirudin. Although the syntheses reactions were performed in our laboratory on an analytical scale, cell-free synthesis in general offers an interesting alternative for the production of active pharmaceutical ingredients. The scalability of cell-free synthesis points out the outstanding potential of this technology and paves the way to future industrial applications.

## Methods

### Sequences and template preparation

The sequence of hirudin-variant 1 (HV1, GenBank Acc. No. KR066903.1) of *Hirudo medicinalis*^60^ was used as the reference. Expression vectors contain the coding sequence of hirudin, the internal ribosomal entry site (IRES) of the cricket paralysis virus (CRPV), the T7-promoter and a signal peptide encoding sequence^40,44,58,69^. All templates are based on the pMA backbone and were generated by gene synthesis (GeneArt, Thermo Fischer). Two different templates were designed: the first construct contained the coding sequence of the original signal peptide of HV1 (WT-HV1), while the second contained the signal peptide of honey bee melittin (Mel-HV1)^55^.

### Cell-free and bacterial synthesis of hirudin

Cell-free protein synthesis was performed based on translationally active eukaryotic lysates from CHO-K1 (ECACC 85051005), *Sf* 21 (DSM ACC 119) and K562 (InVivo BioTech GmbH, Hennigsdorf, Germany) cell lines as previously described^40,44,50^. In seeking to find the most suitable lysate system for hirudin synthesis, the batch reaction mode for the cell-free protein synthesis were used. The reaction mixture contained 40% (v/v) of the respective cell lysate (*Sf*21 or CHO or K562), 60 ng/µl plasmid DNA, 20 mmol/l HEPES (pH 7.6), 70 µmol/l amino acids, 1.45 mmol/l ATP, 0.25 mmol/l GTP, 0.25 mmol/l CTP, 0.25 mmol/l UTP, 83 µmol/l m7G(ppp)G-CAP-analoga, 95 mmol/l KOAc, 2.7 mmol/l Mg(OAc)_2_, 20 µmol/l Poly-G Primer, and 1 U/µl T7 RNA Polymerase. To monitor protein quality and quantity, an aliquot of the reaction mixture was supplemented with ^14^C-labelled leucine (310.0 mCi/mmol, Perkin Elmer, Germany). After preparing the reaction mixture, the volume for the incubation step was divided into aliquots of 50 µl each. Protein synthesis reactions were incubated at 27 °C (*Sf*21 lysate) or at 30 °C (CHO, K562) for 3 h and at 500 rpm (Thermomixer comfort, Eppendorf, Hamburg, Germany). A reaction mixture without plasmid (no-template control, NTC) was prepared as the negative control. After incubation, the ^14^C-leucine labelled reaction mixtures were frozen at − 20 °C until radiometric analysis.

The bacterial hirudin was expressed in *E. coli*. While the exact methods are already described in other places^48,60^. An additional hexahistidin-tag was fused to hirudin for purification, afterwards the tag was removed completely removed due to a factor-Xa cleavage-site and a digest with factor-Xa. The final hirudin was dialysed and kept in elution buffer (20 mmol/l Tris/HCl, 100 mmol/l NaCl, 2 mmol/l CaCl2, pH8.0).

### Preparation of SN2 fraction harbouring the translocated hirudin

The reaction mixtures were fractionated after completion of protein synthesis into TM (total reaction mixture), SN1 (supernatant-1) and MF1 (microsomal fraction). For fractionation, the total reaction mixture was centrifuged for 10 min at 16,000×*g* and 4 °C (5415R microcentrifuge, Eppendorf, Hamburg, Germany). The supernatant-1 (SN1) was separated and the pellet was re-suspended with PBS (pH 7.4) to obtain the MF1. To release translocated hirudin from the lumen of the microsomes, MF1 was treated with 0.02% (w/v) DDM (*n*-Dodecyl-β-d-maltoside) in elution buffer and incubated for 1 h at 25 °C. An additional centrifugation step at 16,000×*g*, 4 °C and 10 min was performed to obtain the released protein in the SN2 (supernatant-2)^40^. The SN2 fraction was separated and used for further studies.

### Analysis of ^14^C-labeled proteins

The total yield (TM), soluble protein (SN1), microsomal fraction (MF1), translocated and released protein (SN2) were determined by hot trichloroacetic acid (TCA)-precipitation. Aliquots of 5 μl of each fraction were taken from the solution, mixed with 3 ml TCA (10% solution with 2% casein hydrolysate) and incubated in a water bath for 15 min, followed by incubation on ice for 30 min. The TCA-precipitated proteins were collected on filters (MN GF-3, Machery-Nagel, Düren, Germany) and washed with 5% TCA using a vacuum filtration system (Hoefer Scientific, Holliston, MA, USA) to remove non-incorporated ^14^C-leucin from the protein solution. Protein loaded filters were placed in scintillation vials and 3 ml scintillation cocktail (Quicksafe A, Zinsser Analytic, Eschborn, Germany) was added. The incorporation of ^14^C-leucine into cell-free expressed hirudin was measured by liquid scintillation counting by double determination (n = 2) using the LS6500 Multi Purpose Scintillation Counter (Beckmann Coulter, Krefeld, Germany).

To determine the homogeneity and molecular size of cell-free synthesized hirudin, 5 µl aliquots of the corresponding fractions were precipitated with 150 µl ice-cold acetone and the samples were incubated on ice for 15 min. Subsequently the samples were centrifuged at 16,000×*g* for 5 min at 4 °C. Protein pellets were re-suspended in 20 µl sample buffer (LDS-sample buffer, Life Technologies, Thermo Fischer, Schwerte, Germany), incubated for 15 min at RT with gentle shaking and heated for 10 min at 70 °C. Samples were loaded onto precast SDS-PAGE gels (10% Bis–Tris NuPAGE Novex gel, Life Technologies) and run for 35 min at 200 V. After electrophoresis, the gels were stained with Coomassie Blue (SimplyBlue SafeStain, Life Technologies), dried for 1 h on Whatman paper at 70 °C (Unigeldryer 3545, Uniequip, Planegg, Germany) and exposed to storage phosphor screens (Mounted GP, GE Healthcare, Solingen, Germany). Radioactively labelled proteins were visualized using a phosphor imager (Typhoon Trio + , GE Healthcare). The determination of radioactively labelled proteins is based on our previous works^44,55,70^.

### Coagulation assays

To verify the anti-coagulatory activity of the different HV1 preparations, two blood coagulation assays were performed: the activated partial thromboplastin time test (aPTT; reference range 22.7–28.9 s) and the thrombin time test (TT; reference range 16.8–21.4 s) using a BFT II analyzer (Siemens Healthcare, Erlangen, Germany). All steps followed the instructions outlined by the manufacturer and were described in detail in our previous works with small deviations^48,71^. 10 µl of the respective SN2 fraction of the cell-free expressed protein were directly transferred into the cuvette immediately before the plasma was added. Dade Ci-Trol 1 (Siemens Healthcare, Erlangen, Germany) was used as standardized human plasma. The incubation of reaction mixtures was carried out at 37.4 °C. Measurements that lasted up to 300 s were stopped and declared as a complete inhibition of clot formation. Clot formation was additionally controlled by eye to exclude technical errors. All samples where measured within 48 h after cell-free synthesis. During this time period all samples were kept at 4 °C to avoid freeze–thaw circles. HV1 expressed in bacteria was kept in aliquots and thawed on ice immediately before the measurements.

Samples were either used directly or diluted in 0.02% (w/v) DDM-solution to adjust the final concentration.

### Immunoblotting

Immunoblotting was performed to evaluate whether or not the single tyrosine residue at position 63 (Tyr-63) in cell-free synthesized HV1 is sulfated. 10 µl of undiluted SN2 fraction which contains either the protein or the background control (NTC) were denatured by mixing with 3 × SDS-sample buffer (0.27 mol/l Tris, 60% (v/v) glycerol, 12% (v/v) β-Mercaptoethanol, 3% (w/v) SDS, 0.6% (w/v) bromophenol blue) and incubation for 10 min at 105 °C. Samples were cooled down and subsequently loaded on self-casted 20% polyacrylamide gels twice in a mirrored arrangement. After the run at 60 V for 4 h in a Mini-PROTEAN Tetra Cell (BioRad, Feldkirchen, Germany), proteins were blotted from the gels onto Roti-NC nitrocellulose membrane (Roth, Karlsruhe, Germany) using a Trans-Blot SD semi-dry transfer cell (BioRad, Feldkirchen, Germany) at 400 mA for 15 min.

The membranes were cut into two halves in a way, that each side contained the same arrangement of samples. Both halves were first blocked with 3% milk-TTBS, and one half was subsequently incubated over night at 4 °C with 10 µl of an anti-sulfotyrosine antibody [Sulfo-1C-A2] (Abcam, Cambridge, UK) whereas the other was incubated with 10 µl of an anti-hirudin antibody [2D7] (Abcam, Cambridge, UK). An anti-mouse IgG HRP-linked antibody (Cell Signaling Technology, Frankfurt /M., Germany) was used as the secondary antibody^72^. Blots were incubated with WesternBright ECL HRP reagent (Biozym, Hessisch Oldendorf, Germany) and the bands were detected using a ChemoStar ECL & Fluorescence Imager (Intas Science Imaging Instruments, Göttingen, Germany).

### MALDI-TOF

Samples for MALDI-TOF mass spectrometric analysis were prepared using HyperSep-C18-tips (Thermo Scientific, Schwerte, Germany) according to the manufacturer’s recommendations. Sample application was performed via dried-droplet on MSP 96 target ground steel (Bruker Daltonics, Bremen, Germany) with 1 µl sinapinic-matrix (45 mmol/l sinapinic acid in 70% (v/v) H_2_O, 30% (v/v) acetonitrile, 0.3% (v/v) trifluoroacetic acid). After drying under vacuum, samples were analyzed using the LP-mode and 60–90% laser intensity by a Microflex device (Bruker Daltonics, Bremen, Germany). The free software mMass v. 5.5.0 was used to evaluate the mass spectra^73^.

### Ethical approval

We declare that the experiments described in this paper comply with the current laws in Germany. This article does not contain any studies with human participants performed by any of the authors.

## Supplementary information

Supplementary Information.
