# Supplementary material for: Cell-free synthesis of the hirudin variant 1 of the blood-sucking leech Hirudo medicinalis
Source: Sci Rep. 2020 Nov 13;10:19818. doi: 10.1038/s41598-020-76715-w (PMC7666225; doi:10.1038/s41598-020-76715-w)
Supplement: Supplementary file 1 — Supplementary Information. [file 41598_2020_76715_MOESM1_ESM.pdf]

Supplementary Material

Cell-free synthesis of the hirudin variant 1 of the blood-sucking leech Hirudo medicinalis

Doreen A. Wüstenhagen[1,+], Phil Lukas[2,+], Christian Müller[2], Simone A. Aubele[1], Jan-Peter Hildebrandt[2], Stefan Kubick[1,3,*]

[1]Fraunhofer Institute for Cell Therapy and Immunology (IZI), Branch Bioanalytics and Bioprocesses Potsdam-Golm (IZI-BB), 14476, Germany
[2]Animal Physiology and Biochemistry, Zoological Institute and Museum, University of Greifswald, Greifswald, 17489, Germany
[3]Faculty of Health Sciences, Joint Faculty of the Brandenburg University of Technology Cottbus – Senftenberg, the Brandenburg Medical School Theodor Fontane and the University of Potsdam, Neuruppin, 16816, Germany

+ Both authors contributed equally to this work
* Corresponding author; email: Stefan.kubick@izi-bb.fraunhofer.de; phone: +49 331 58187-306

Besides the SN2-fraction clotting times in presence of SN1-fraction were determined by double determination as well. Besides the higher concentration within the SN1 fraction compared to the SN2-fraction, the influence of the clotting time by the SN1-fraction seem to be lesser then those of the SN2-fraction.

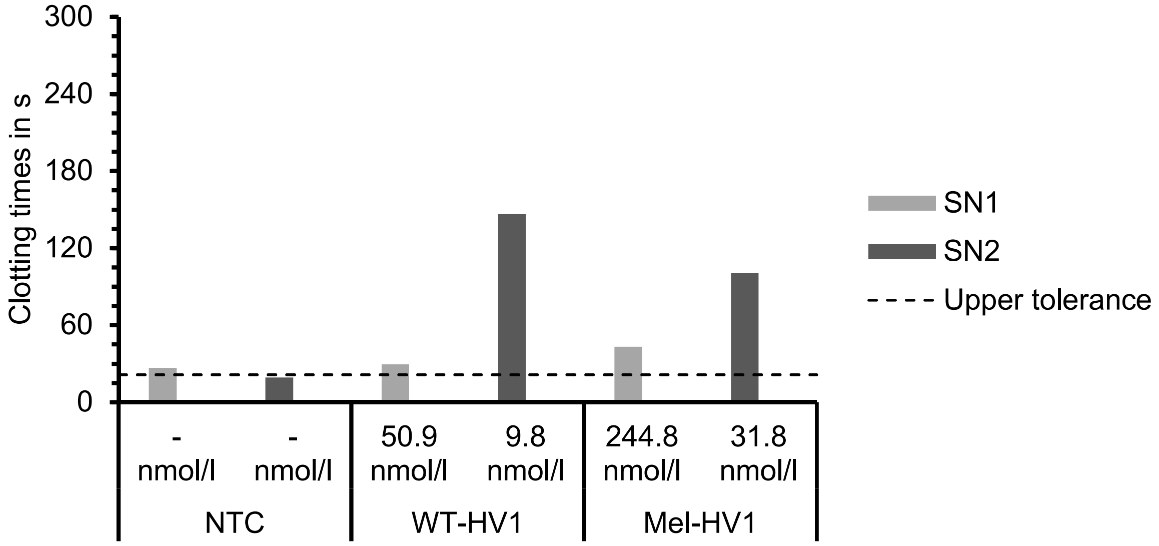

Supplementary figure 1: Anti-coagulatory activities of SN1 and SN2 fractions containing no template control (NTC), WT-HV1 and Mel-HV1 synthesized in the K562 cell-free system as determined by the thrombin time test assay. The SN1 fractions contained unprocessed protein including the signal sequence, while the SN2 fractions contained the final secreted protein without signal peptide (see preparation of SN2 in methods section). Final concentrations of test proteins are given in nmol/l. (n = 2)

Images for Western Blot (manuscript, fig. 6) were obtained as described in the methods section. rHV1, WT-HV1, Mel-HV1 and the background control NTC were applied together on a protein gel and analyzed on a Western blot. The signals were recorded over shutter times of from 5 s to 4 min 10 s. The intensity of the signals from rHV1 are significantly stronger than those from WT-HV1 and Mel-HV1. To avoid overexposure and to show all results in one image rHV1 was displayed with 5 s and WT-HV1 and Mel-HV1 with a shutter time of 50 s. No subsequent changes in contrast were made, only raw data was used and displayed. PDF-files were created based on the raw files. Corresponding images were extracted from the PDF using the online tool (https://tools.pdf24.org/de/pdf-seiten-extrahieren). Afterwards the PDF was converted to a PNG image with 600 dpi using the same online tool (https://tools.pdf24.org/de/pdf-in-bilder). The final version was excised and assembled using Microsoft Office Home and Student 2016. Therefore the image of the same gel was used with different shutter times to avoid overexposure for recombinant HV1 on the one hand and fade bands for the cell free derived HV1 on the other. A complete time-lapse of all images is given below from 5 s to 4 min 10 s shutter time.

2019-08-04 HV1 zell vs bac,1-1. scan_raw.tif

Scan Date:	2019-08-04 11:22:38
Image Name:	1. scan
Method:	Chemiluminescence
Scan Mode:	list increment
Scan Number:	1. scan
Temperature:	OK
EmissionFilter:	Open
Shutter Time:	5 sec
Binning:	1x1

Range: 0,0% - 100,0%, Gamma: 1,000

2019-08-04 HV1 zell vs bac,2-1. scan+Marker_raw.tif

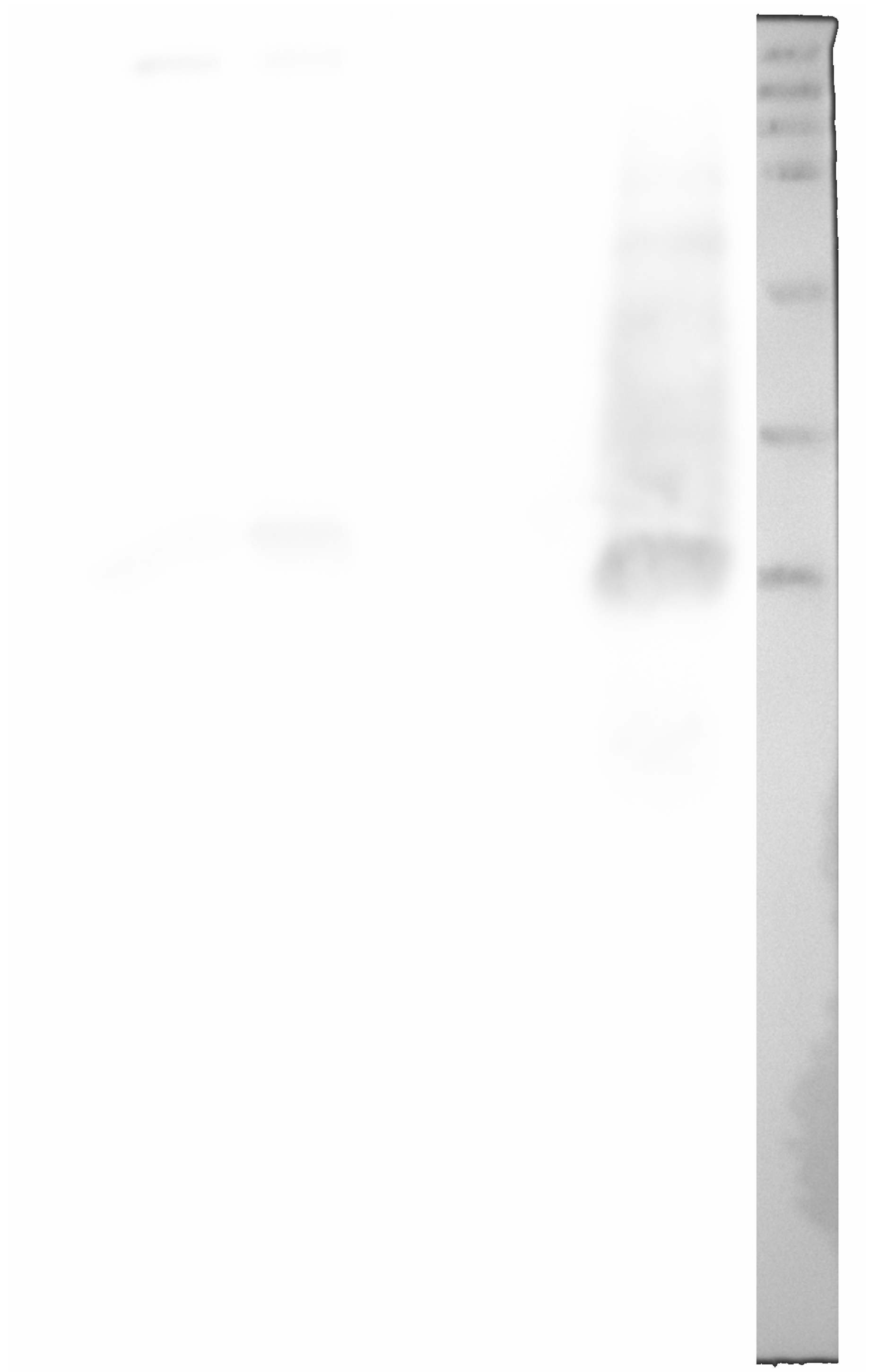

Scan Date:	2019-08-04 11:22:38
Image Name:	1. scan+Marker
Method:	Chemiluminescence
Scan Mode:	list increment
Scan Number:	1. scan+marker
Temperature:	OK
EmissionFilter:	Open
Shutter Time:	5 sec
Binning:	1x1

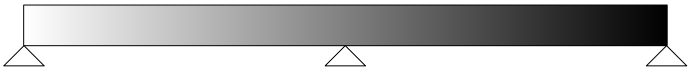

Range: 0,0% - 100,0%, Gamma: 1,000

2019-08-04 HV1 zell vs bac,3-2. scan_raw.tif

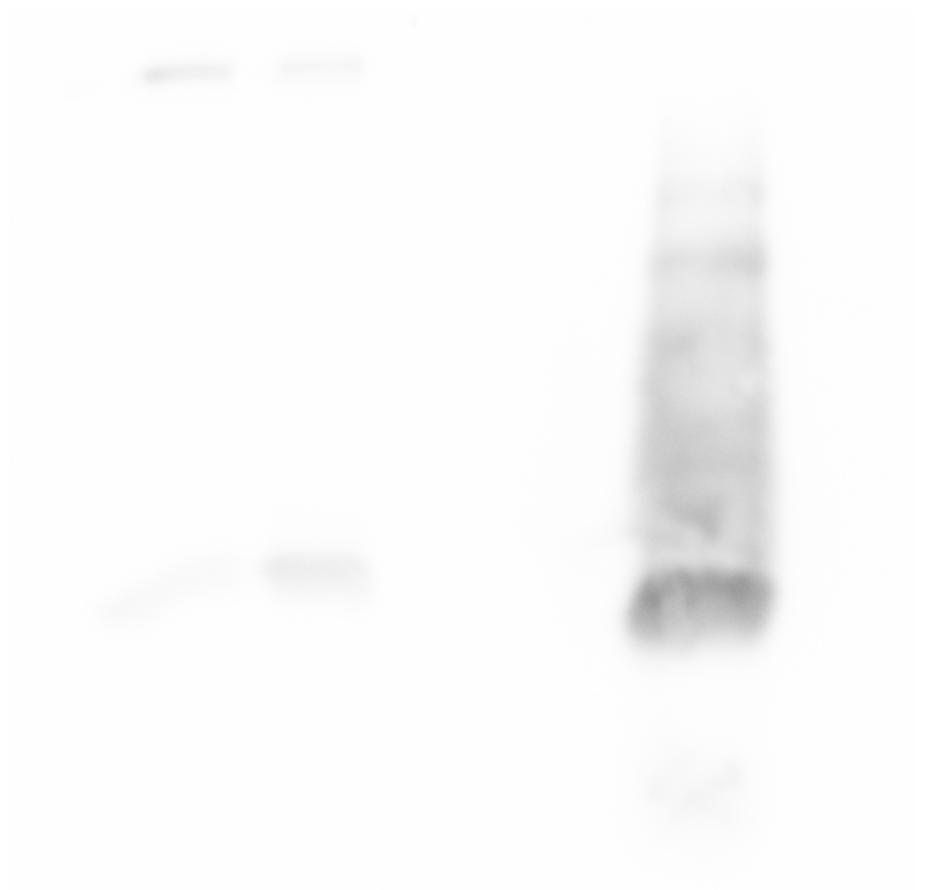

Scan Date:	2019-08-04 11:22:38
Image Name:	2. scan
Method:	Chemiluminescence
Scan Mode:	list increment
Scan Number:	2. scan
Temperature:	OK
EmissionFilter:	Open
Shutter Time:	10 sec
Binning:	1x1

Range: 0,0% - 100,0%, Gamma: 1,000

2019-08-04 HV1 zell vs bac,4-2. scan+Marker_raw.tif

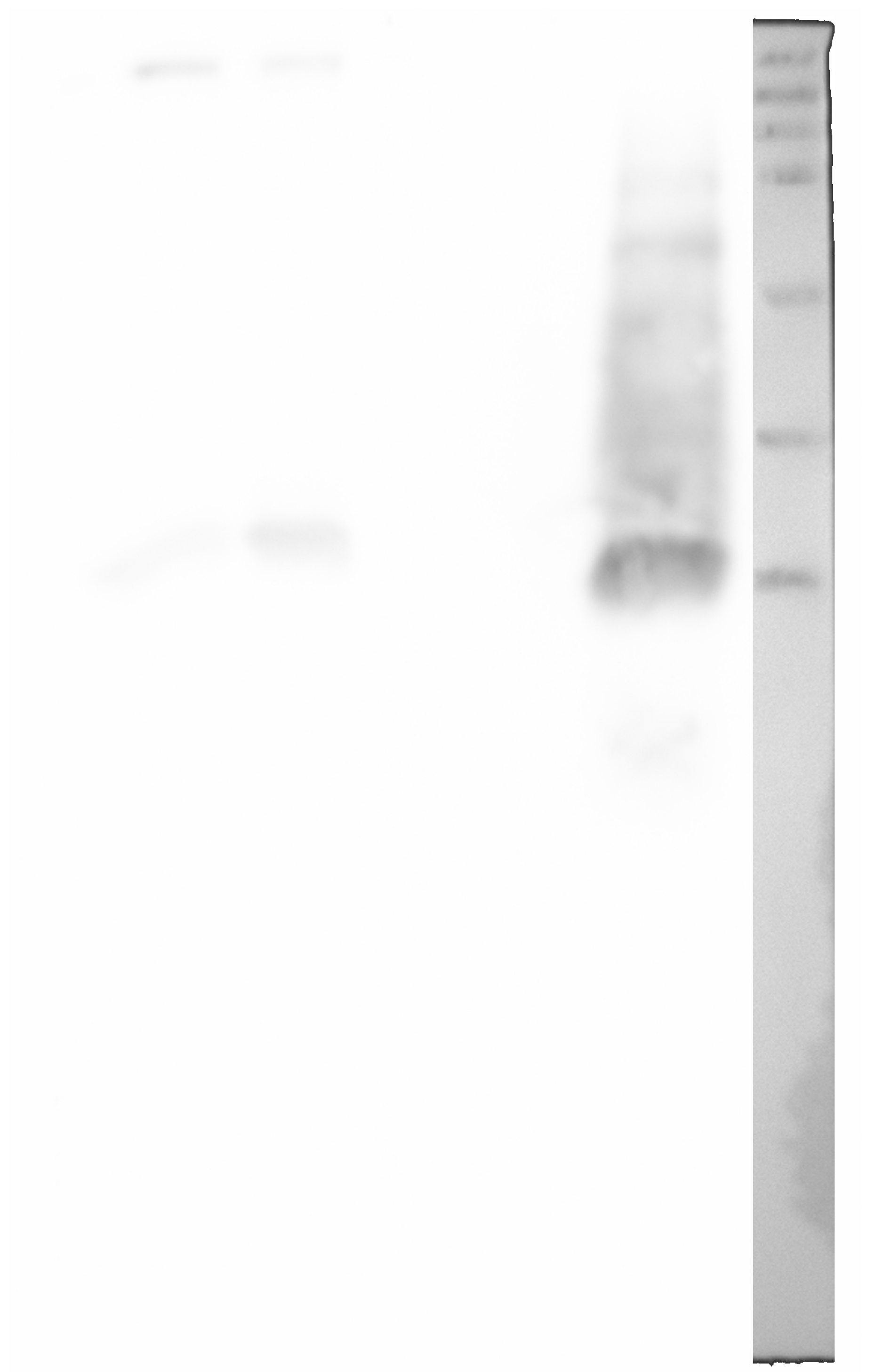

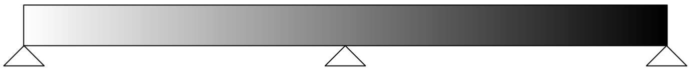

Range: 0,0% - 100,0%, Gamma: 1,000

Scan Date:	2019-08-04 11:22:38
Image Name:	2. scan+Marker
Method:	Chemiluminescence
Scan Mode:	list increment
Scan Number:	2. scan+marker
Temperature:	OK
EmissionFilter:	Open
Shutter Time:	10 sec
Binning:	1x1

2019-08-04 HV1 zell vs bac,5-3. scan_raw.tif

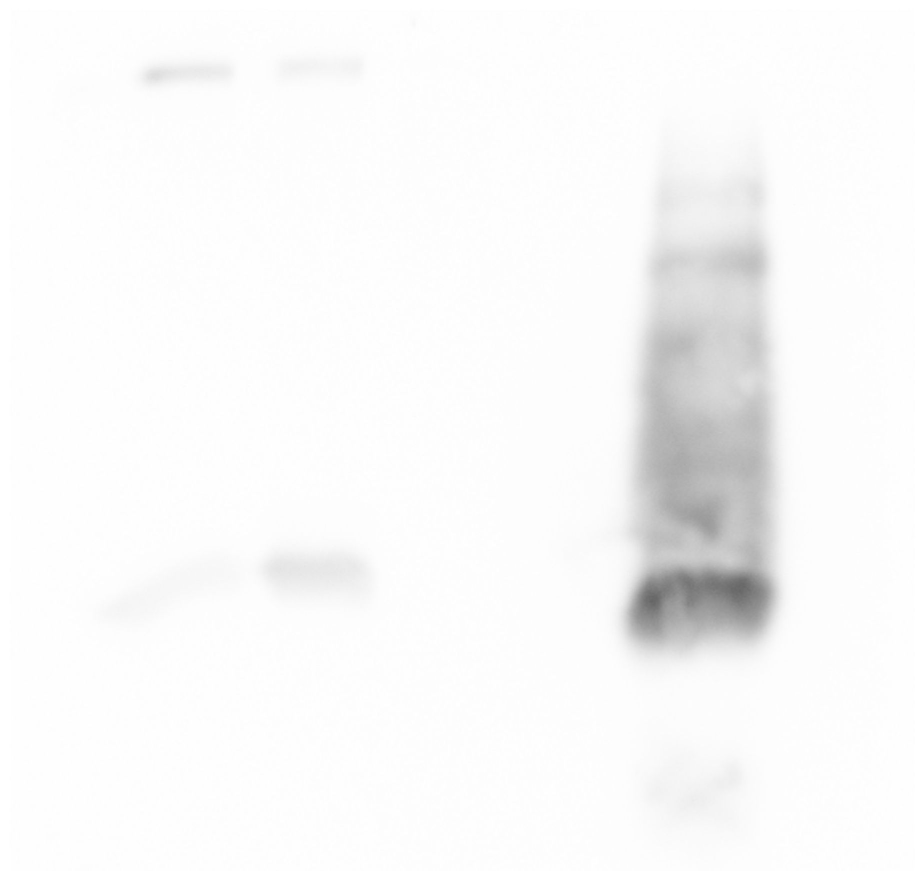

Scan Date:	2019-08-04 11:22:38
Image Name:	3. scan
Method:	Chemiluminescence
Scan Mode:	list increment
Scan Number:	3. scan
Temperature:	OK
EmissionFilter:	Open
Shutter Time:	15 sec
Binning:	1x1

Range: 0,0% - 100,0%, Gamma: 1,000

2019-08-04 HV1 zell vs bac,6-3. scan+Marker_raw.tif

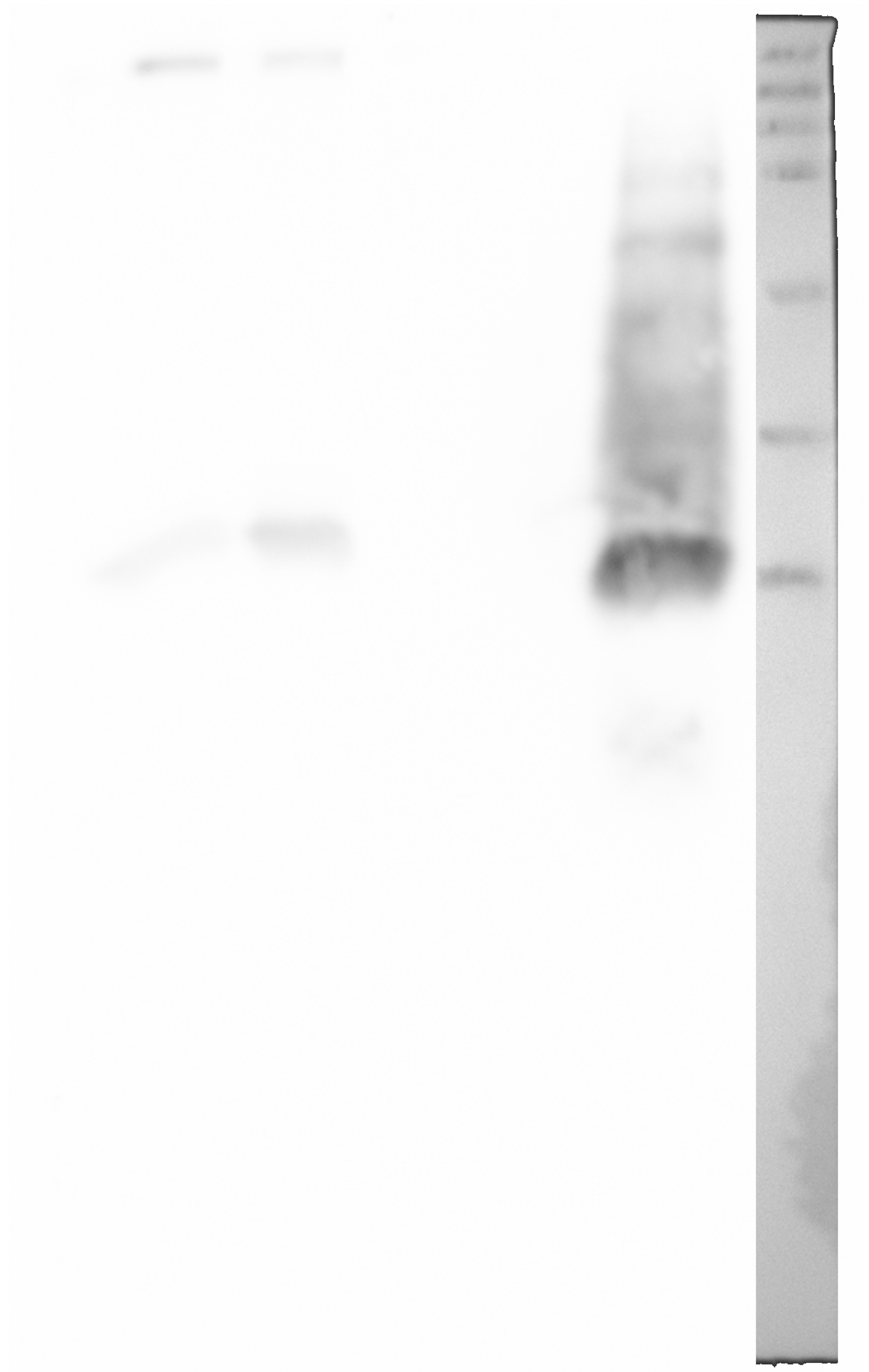

Scan Date:	2019-08-04 11:22:38
Image Name:	3. scan+Marker
Method:	Chemiluminescence
Scan Mode:	list increment
Scan Number:	3. scan+marker
Temperature:	OK
EmissionFilter:	Open
Shutter Time:	15 sec
Binning:	1x1

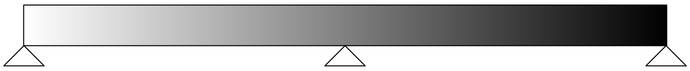

Range: 0,0% - 100,0%, Gamma: 1,000

2019-08-04 HV1 zell vs bac,7-4. scan_raw.tif

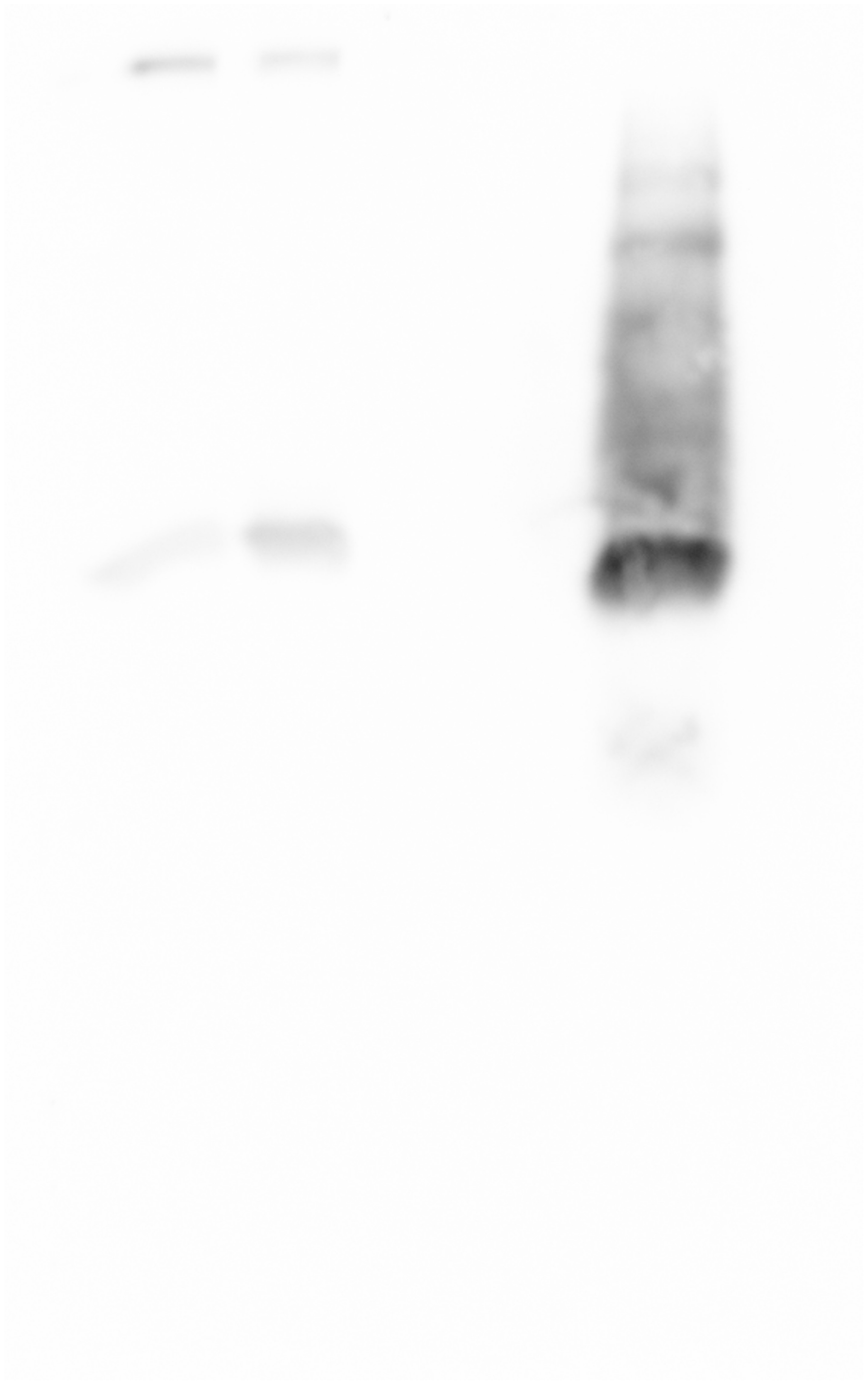

Scan Date:	2019-08-04 11:22:38	
Image Name:	4. scan	
Method:	Chemiluminescence	Range: 0,0% - 100,0%, Gamma: 1,000
Scan Mode:	list increment	
Scan Number:	4. scan	
Temperature:	OK	
EmissionFilter:	Open	
Shutter Time:	20 sec	
Binning:	1x1	

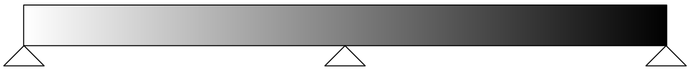

2019-08-04 HV1 zell vs bac,8-4. scan+Marker_raw.tif

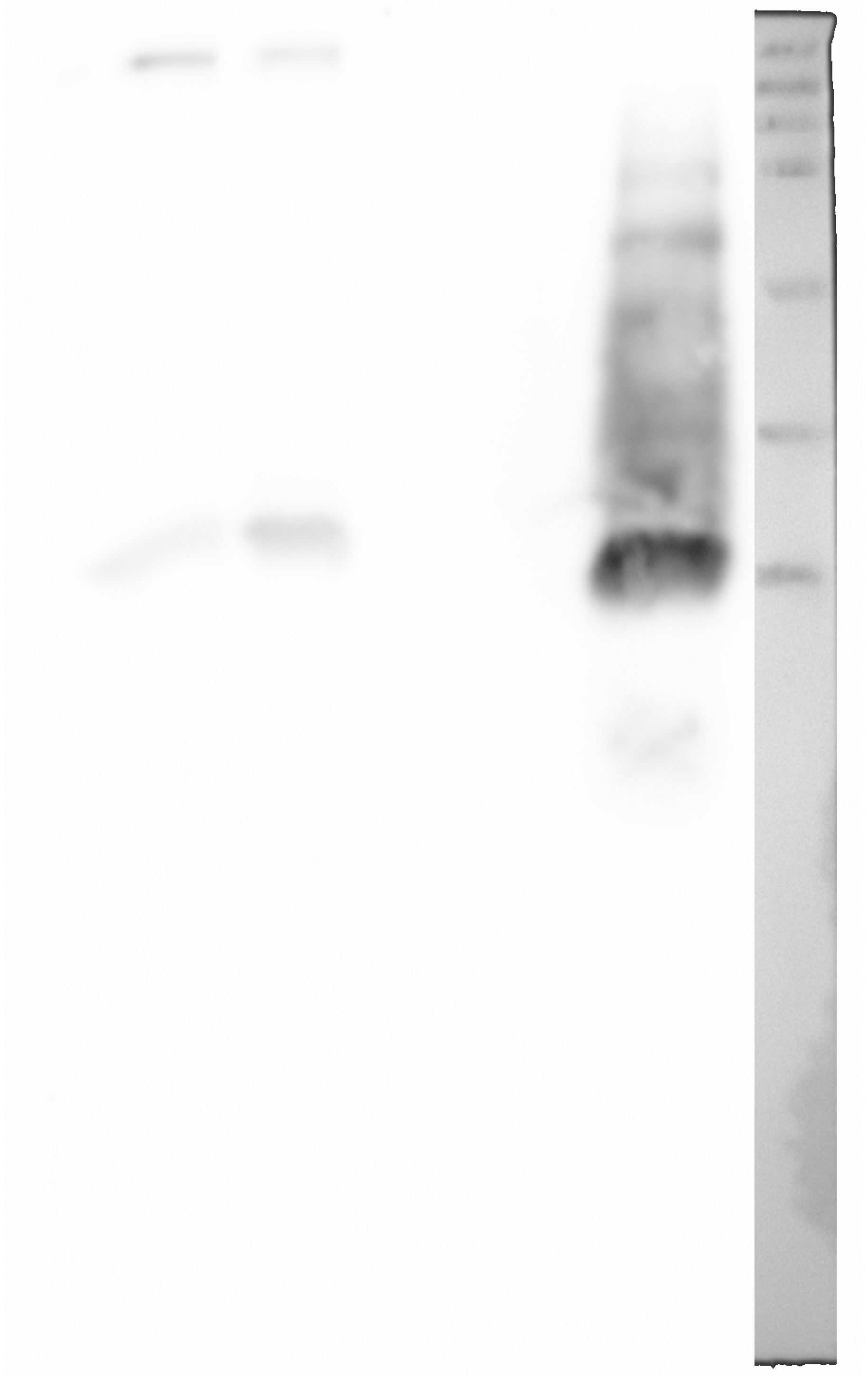

Scan Date:	2019-08-04 11:22:38
Image Name:	4. scan+Marker
Method:	Chemiluminescence
Scan Mode:	list increment
Scan Number:	4. scan+marker
Temperature:	OK
EmissionFilter:	Open
Shutter Time:	20 sec
Binning:	1x1

Range: 0,0% - 100,0%, Gamma: 1,000

2019-08-04 HV1 zell vs bac,9-5. scan_raw.tif

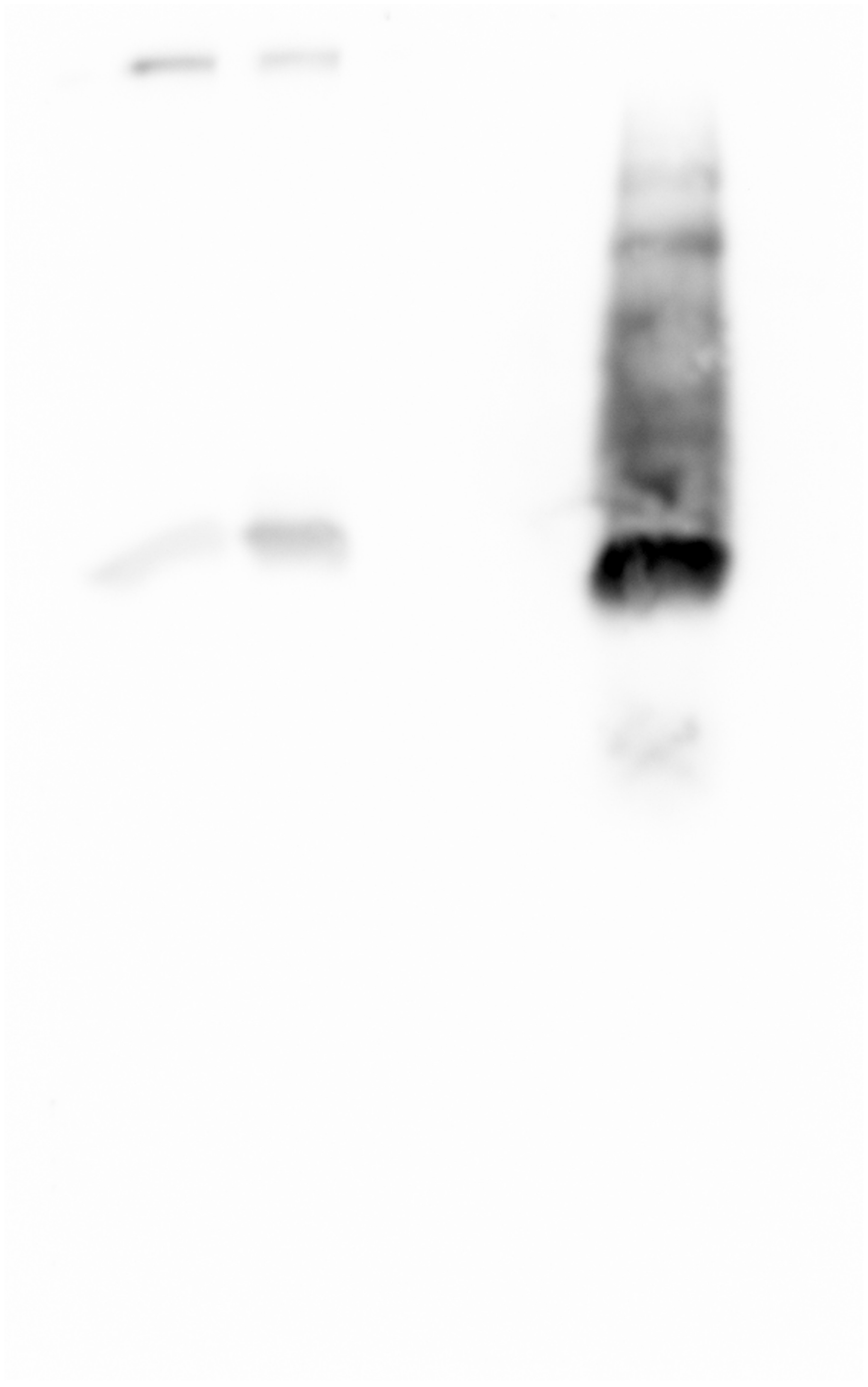

Scan Date:	2019-08-04 11:22:38
Image Name:	5. scan
Method:	Chemiluminescence
Scan Mode:	list increment
Scan Number:	5. scan
Temperature:	OK
EmissionFilter:	Open
Shutter Time:	30 sec
Binning:	1x1

Range: 0,0% - 100,0%, Gamma: 1,000

2019-08-04 HV1 zell vs bac,10-5. scan+Marker_raw.tif

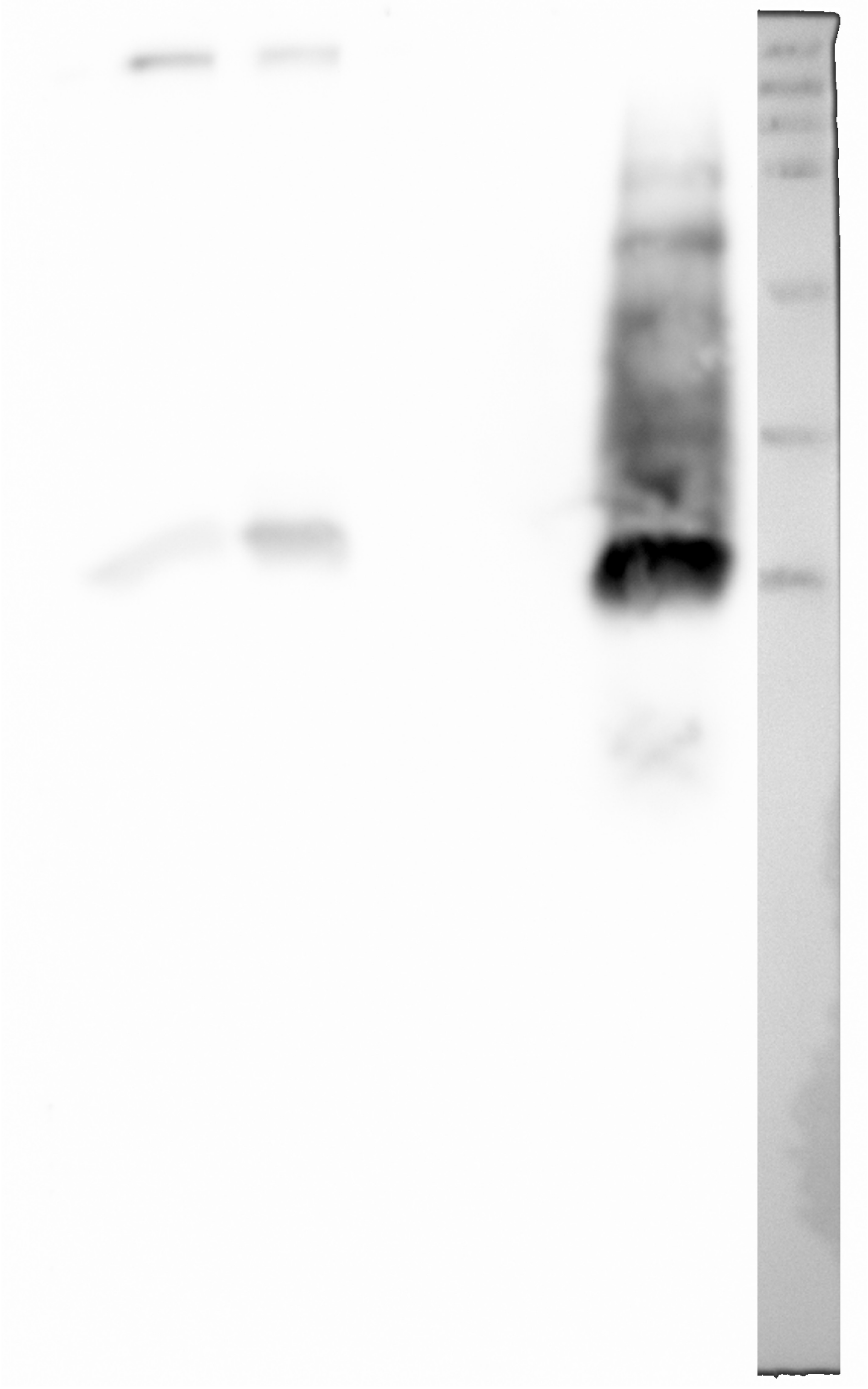

Scan Date:	2019-08-04 11:22:38
Image Name:	5. scan+Marker
Method:	Chemiluminescence
Scan Mode:	list increment
Scan Number:	5. scan+marker
Temperature:	OK
EmissionFilter:	Open
Shutter Time:	30 sec
Binning:	1x1

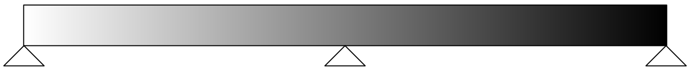

Range: 0,0% - 100,0%, Gamma: 1,000

2019-08-04 HV1 zell vs bac,11-6. scan_raw.tif

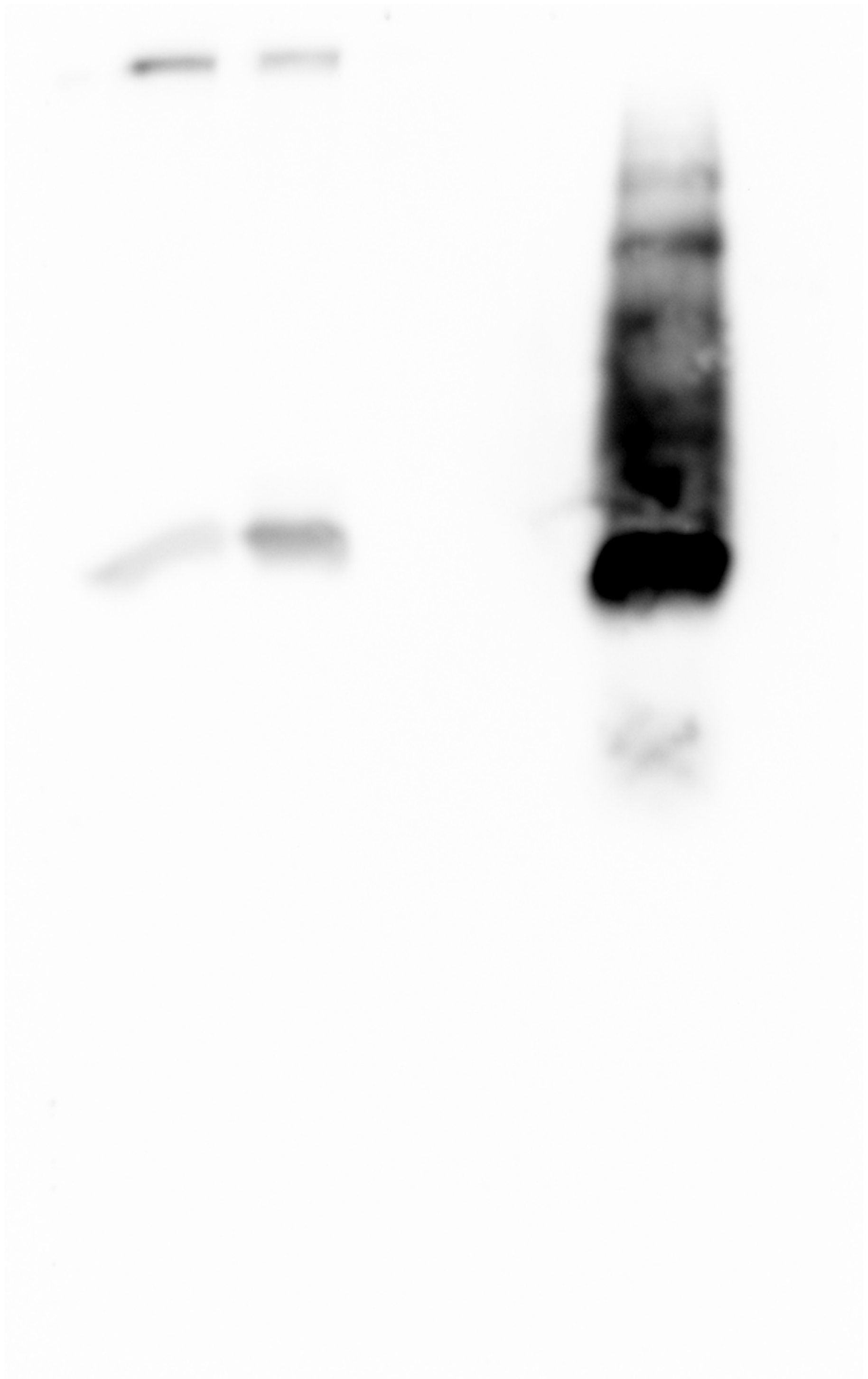

Scan Date:	2019-08-04 11:22:38
Image Name:	6. scan
Method:	Chemiluminescence
Scan Mode:	list increment
Scan Number:	6. scan
Temperature:	OK
EmissionFilter:	Open
Shutter Time:	50 sec
Binning:	1x1

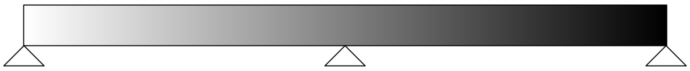

Range: 0,0% - 100,0%, Gamma: 1,000

2019-08-04 HV1 zell vs bac,12-6. scan+Marker_raw.tif

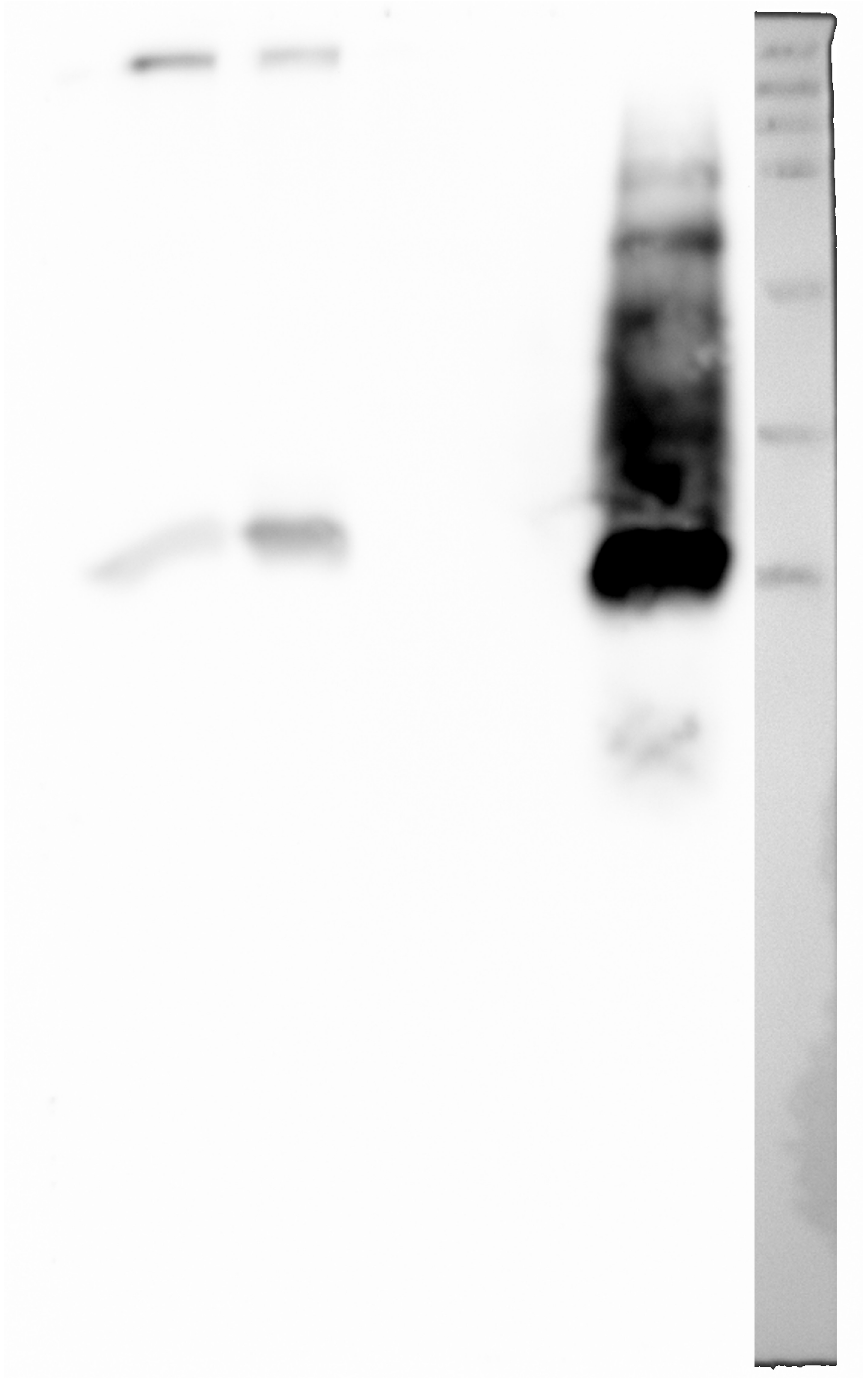

Scan Date:	2019-08-04 11:22:38
Image Name:	6. scan+Marker
Method:	Chemiluminescence
Scan Mode:	list increment
Scan Number:	6. scan+marker
Temperature:	OK
EmissionFilter:	Open
Shutter Time:	50 sec
Binning:	1x1

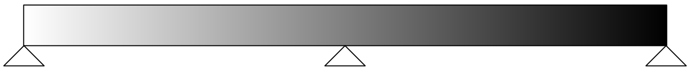

Range: 0,0% - 100,0%, Gamma: 1,000

2019-08-04 HV1 zell vs bac,13-7. scan_raw.tif

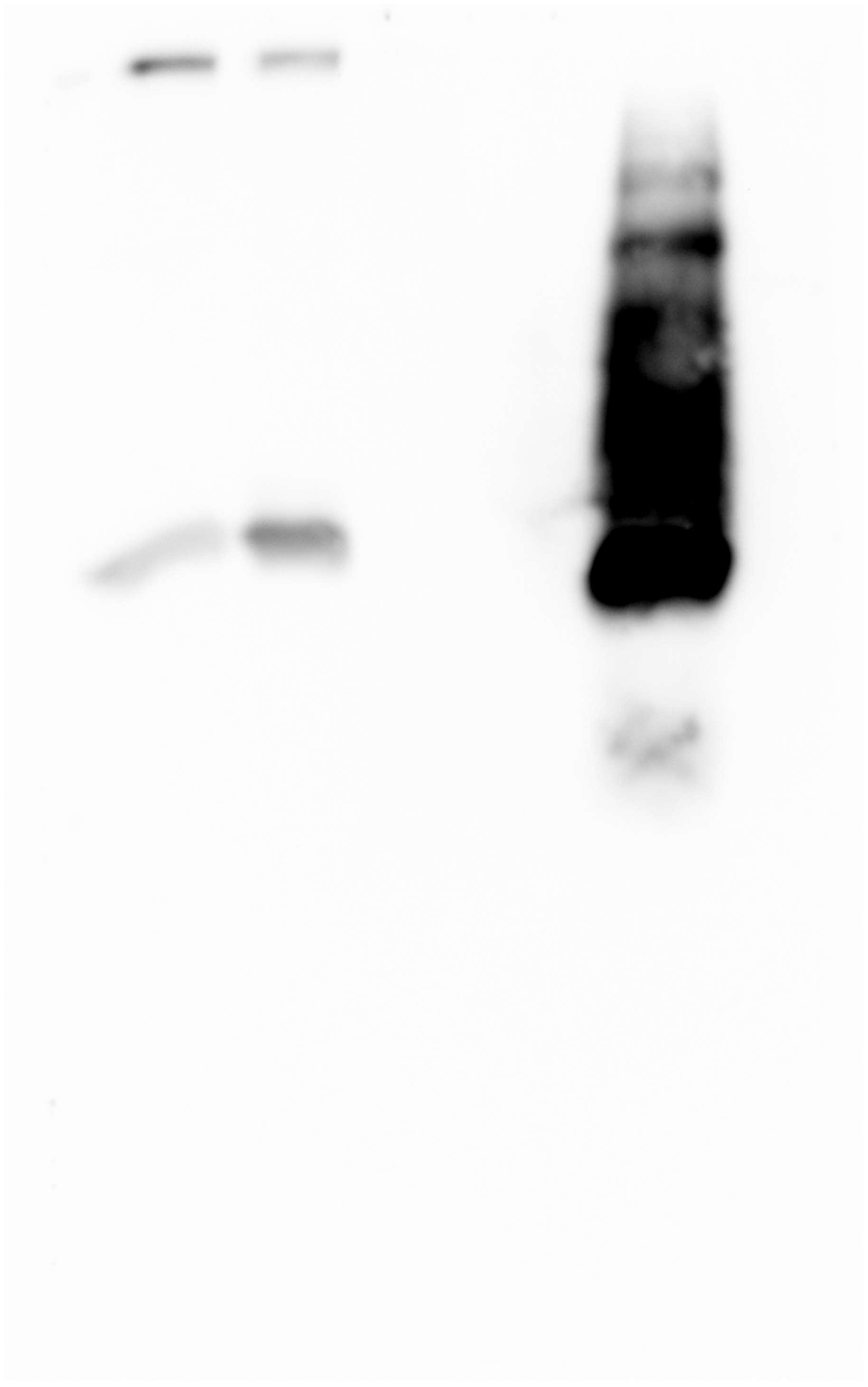

Scan Date:	2019-08-04 11:22:38
Image Name:	7. scan
Method:	Chemiluminescence
Scan Mode:	list increment
Scan Number:	7. scan
Temperature:	OK
EmissionFilter:	Open
Shutter Time:	1:10 min
Binning:	1x1

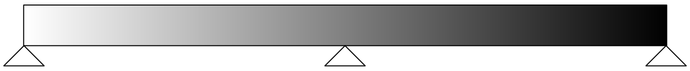

Range: 0,0% - 100,0%, Gamma: 1,000

2019-08-04 HV1 zell vs bac,14-7. scan+Marker_raw.tif

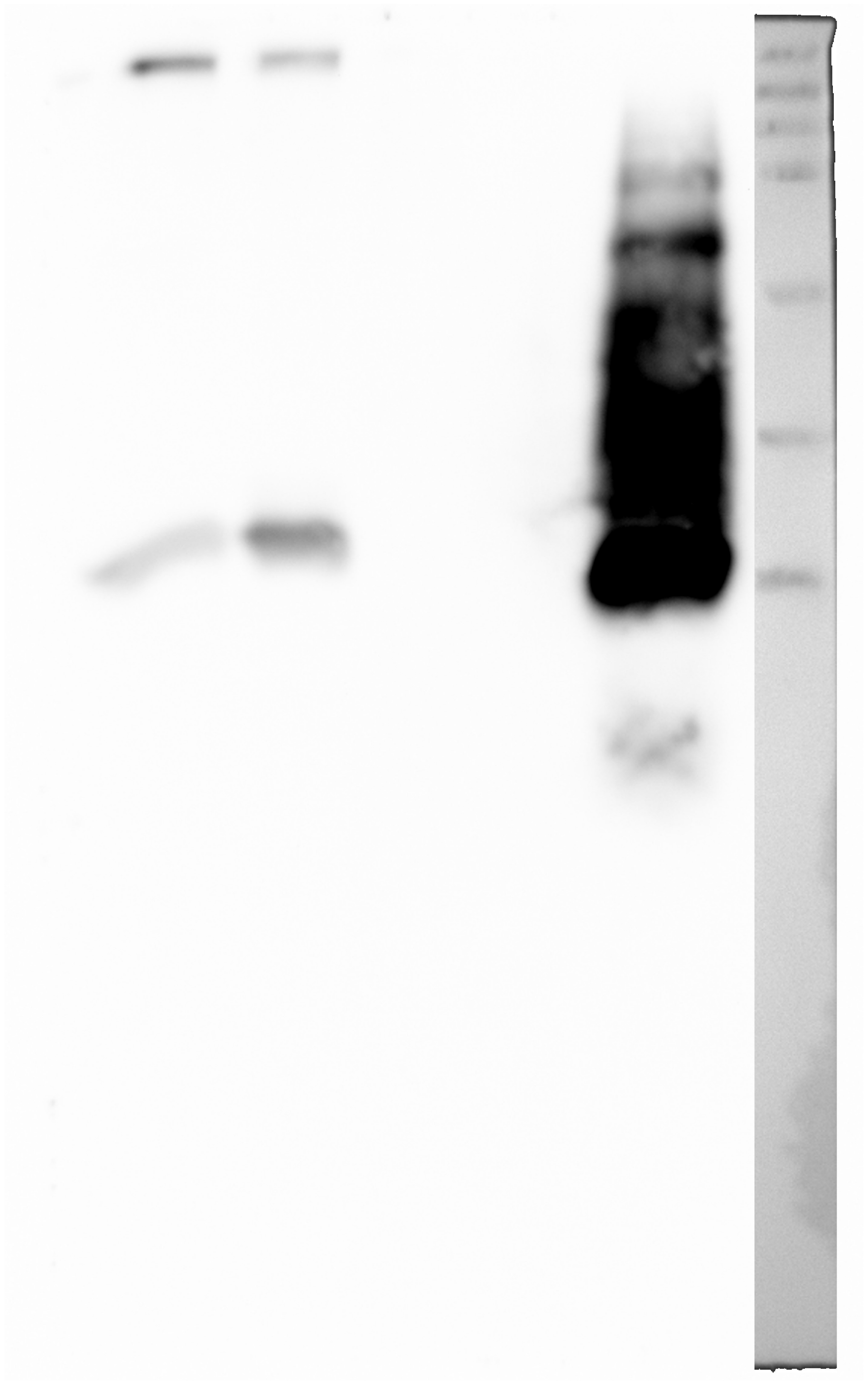

Scan Date:	2019-08-04 11:22:38
Image Name:	7. scan+Marker
Method:	Chemiluminescence
Scan Mode:	list increment
Scan Number:	7. scan+marker
Temperature:	OK
EmissionFilter:	Open
Shutter Time:	1:10 min
Binning:	1x1

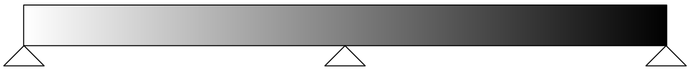

Range: 0,0% - 100,0%, Gamma: 1,000

2019-08-04 HV1 zell vs bac,15-8. scan_raw.tif

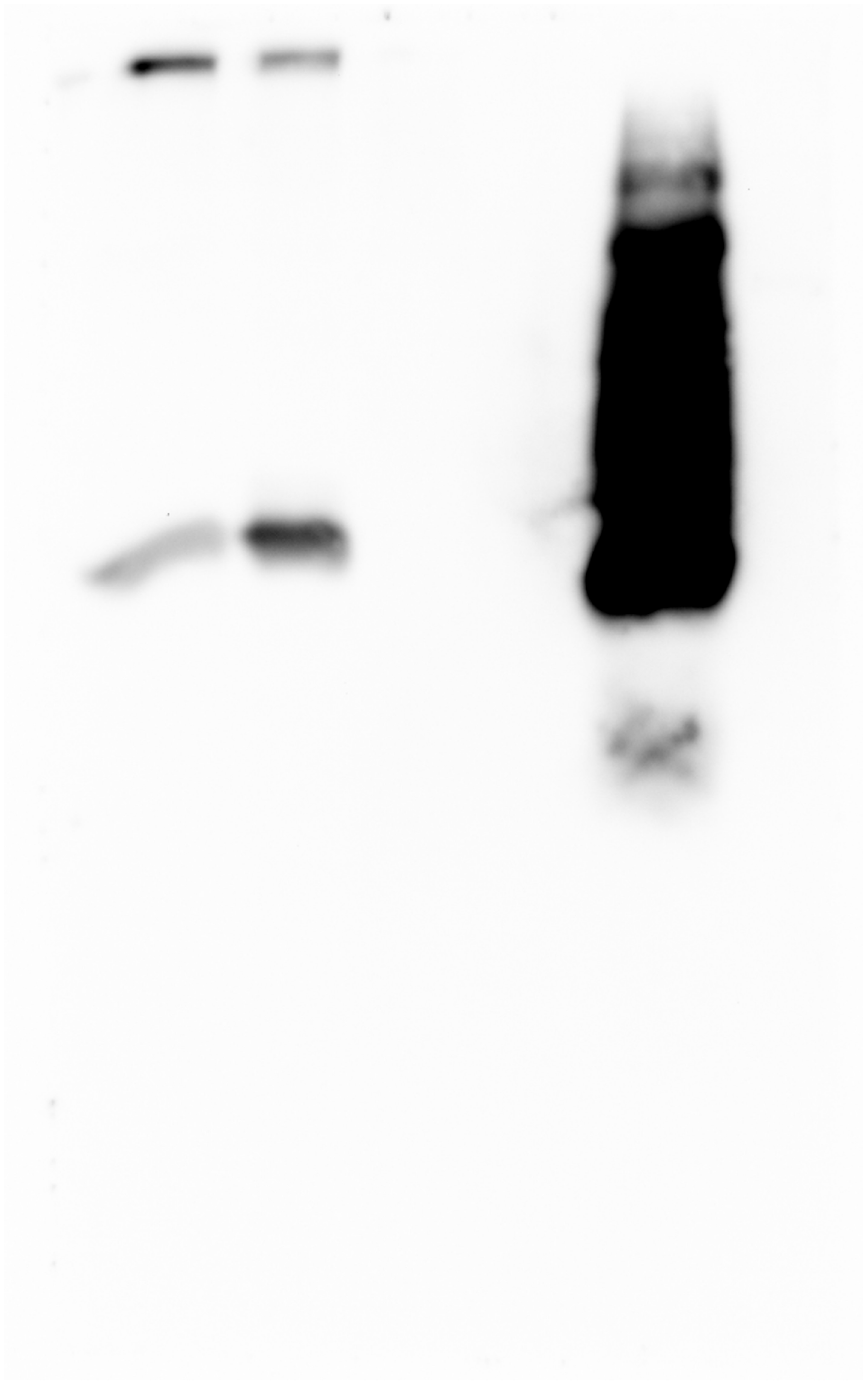

Scan Date:	2019-08-04 11:22:38
Image Name:	8. scan
Method:	Chemiluminescence
Scan Mode:	list increment
Scan Number:	8. scan
Temperature:	OK
EmissionFilter:	Open
Shutter Time:	2:10 min
Binning:	1x1

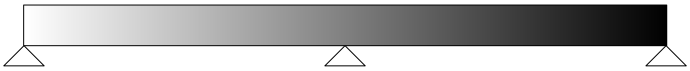

Range: 0,0% - 100,0%, Gamma: 1,000

2019-08-04 HV1 zell vs bac,16-8. scan+Marker_raw.tif

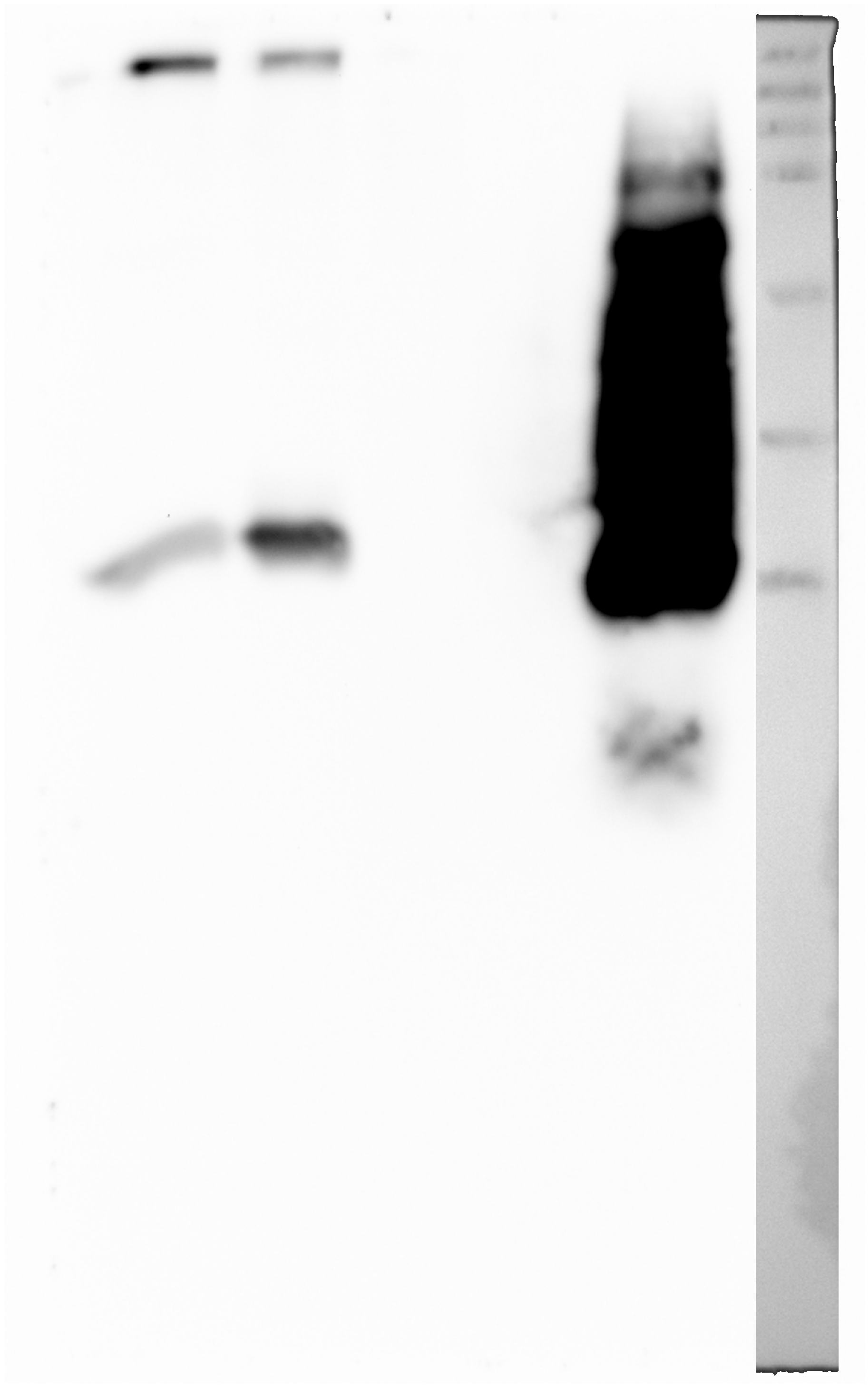

Scan Date: 2019-08-04 11:22:38
Image Name: 8. scan+Marker
Method: Chemiluminescence
Scan Mode: list increment
Scan Number: 8. scan+marker
Temperature: OK
EmissionFilter: Open
Shutter Time: 2:10 min
Binning: 1x1

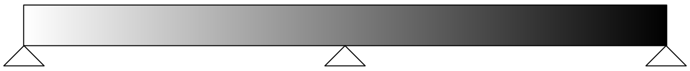

Range: 0,0% - 100,0%, Gamma: 1,000

2019-08-04 HV1 zell vs bac,17-9. scan_raw.tif

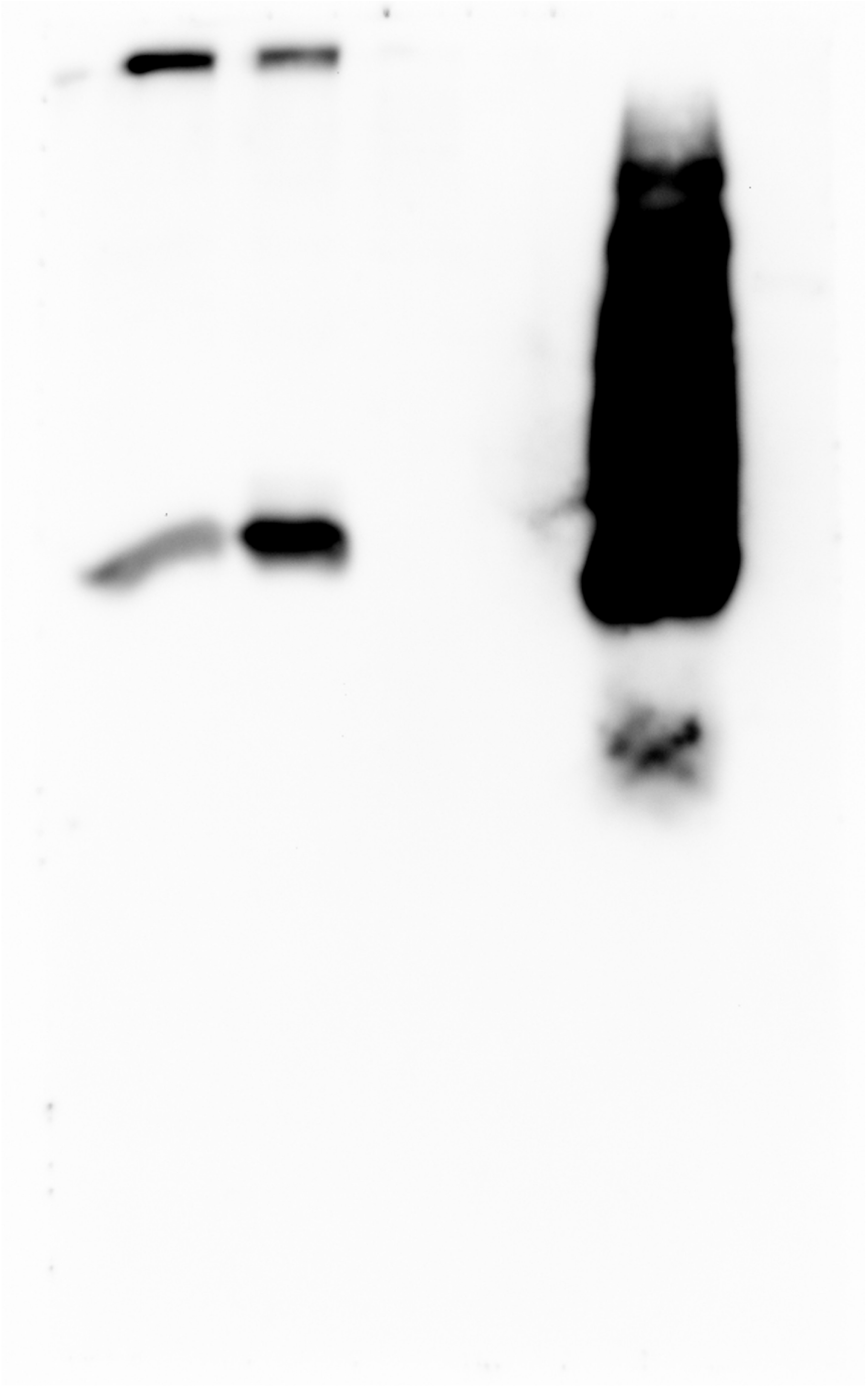

Scan Date:	2019-08-04 11:22:38
Image Name:	9. scan
Method:	Chemiluminescence
Scan Mode:	list increment
Scan Number:	9. scan
Temperature:	OK
EmissionFilter:	Open
Shutter Time:	4:10 min
Binning:	1x1

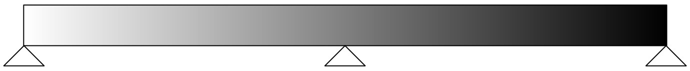

Range: 0,0% - 100,0%, Gamma: 1,000

2019-08-04 HV1 zell vs bac,18-9. scan+Marker_raw.tif

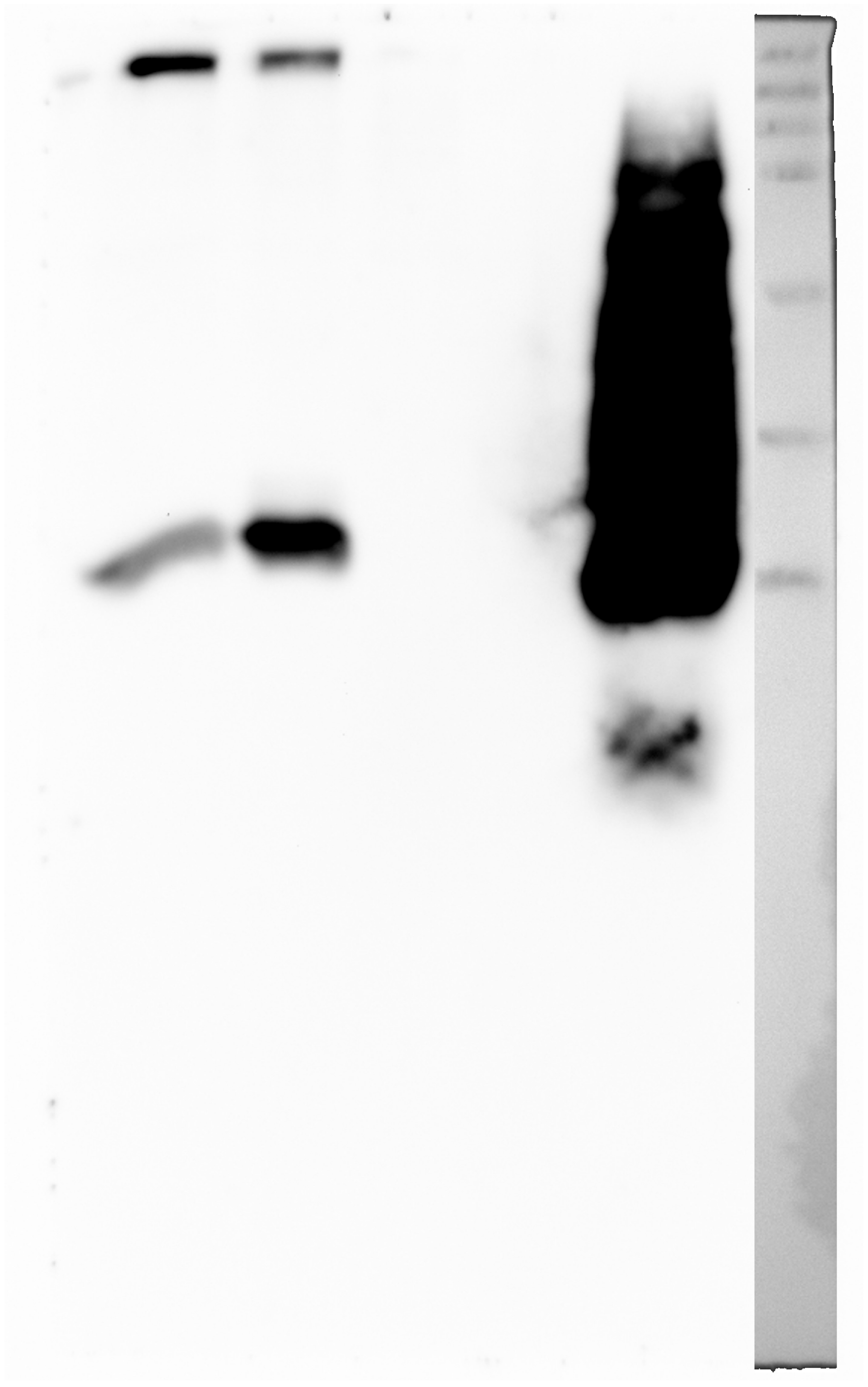

Range: 0,0% - 100,0%, Gamma: 1,000

Scan Date:	2019-08-04 11:22:38
Image Name:	9. scan+Marker
Method:	Chemiluminescence
Scan Mode:	list increment
Scan Number:	9. scan+marker
Temperature:	OK
EmissionFilter:	Open
Shutter Time:	4:10 min
Binning:	1x1

2019-08-04 HV1 zell vs bac,19-Marker_raw.tif

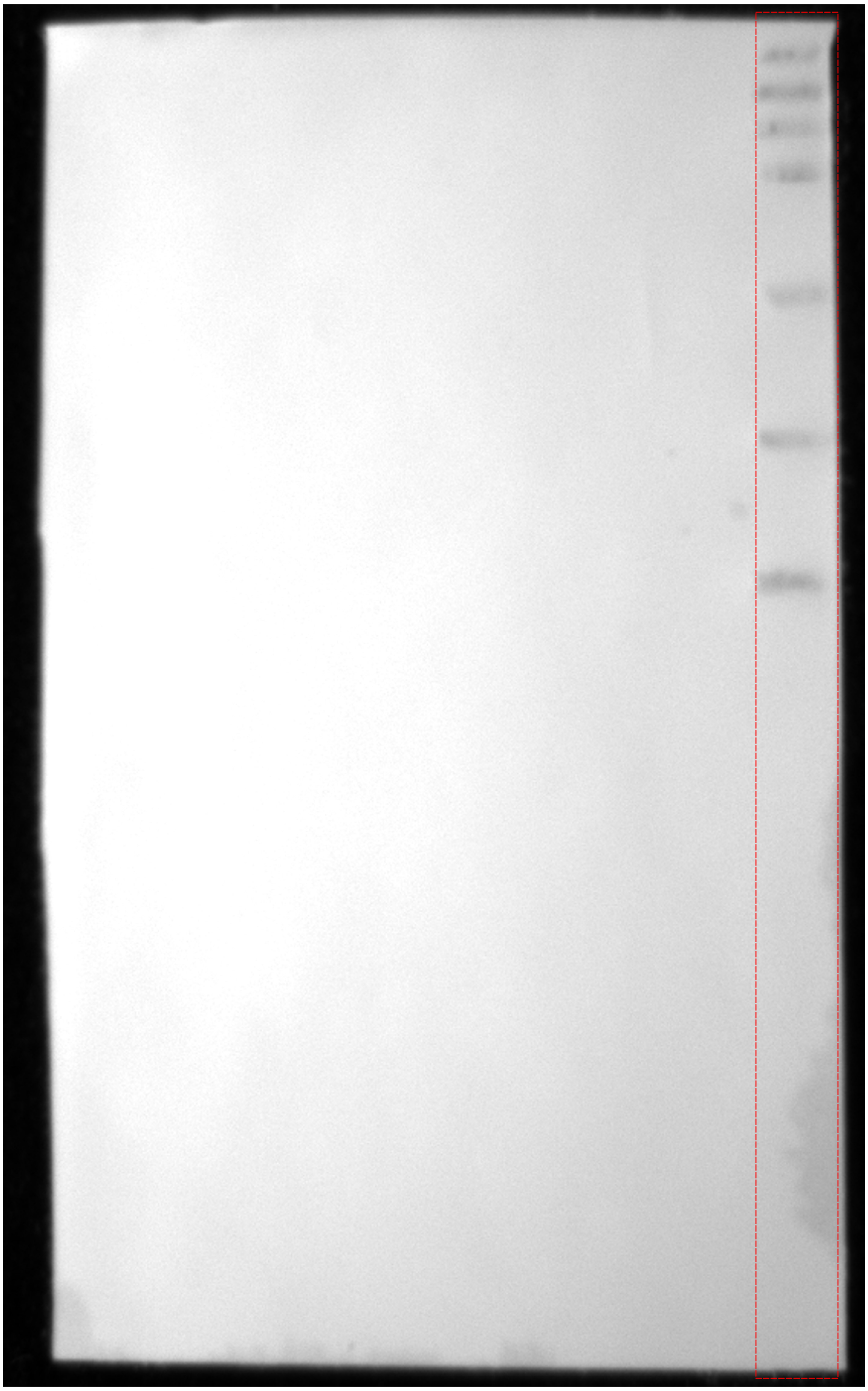

Scan Date: 2019-08-04 11:27:10
Image Name: Marker
Method: Chemiluminescence
Scan Mode: marker
Scan Number: marker
Temperature: OK
Excitation Light: Focus Light
EmissionFilter: Open
Shutter Time: 75 msec
Binning: 1x1

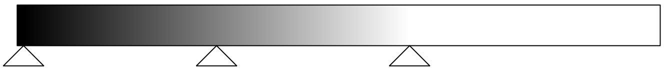

Range: 0,9% - 61,1%, Gamma: 1,000
